# CdiA Effectors from Uropathogenic *Escherichia coli* Use Heterotrimeric Osmoporins as Receptors to Recognize Target Bacteria

**DOI:** 10.1371/journal.ppat.1005925

**Published:** 2016-10-10

**Authors:** Christina M. Beck, Julia L. E. Willett, David A. Cunningham, Jeff J. Kim, David A. Low, Christopher S. Hayes

**Affiliations:** 1 Department of Molecular, Cellular and Developmental Biology, University of California, Santa Barbara, Santa Barbara, California, United States of America; 2 Biomolecular Science and Engineering Program, University of California, University of California, Santa Barbara, Santa Barbara, California, United States of America; University of California Davis School of Medicine, UNITED STATES

## Abstract

Many Gram-negative bacterial pathogens express contact-dependent growth inhibition (CDI) systems that promote cell-cell interaction. CDI^+^ bacteria express surface CdiA effector proteins, which transfer their C-terminal toxin domains into susceptible target cells upon binding to specific receptors. CDI^+^ cells also produce immunity proteins that neutralize the toxin domains delivered from neighboring siblings. Here, we show that CdiA^EC536^ from uropathogenic *Escherichia coli* 536 (EC536) uses OmpC and OmpF as receptors to recognize target bacteria. *E*. *coli* mutants lacking either *ompF* or *ompC* are resistant to CDI^EC536^-mediated growth inhibition, and both porins are required for target-cell adhesion to inhibitors that express CdiA^EC536^. Experiments with single-chain OmpF fusions indicate that the CdiA^EC536^ receptor is heterotrimeric OmpC-OmpF. Because the OmpC and OmpF porins are under selective pressure from bacteriophages and host immune systems, their surface-exposed loops vary between *E*. *coli* isolates. OmpC polymorphism has a significant impact on CDI^EC536^ mediated competition, with many *E*. *coli* isolates expressing alleles that are not recognized by CdiA^EC536^. Analyses of recombinant OmpC chimeras suggest that extracellular loops L4 and L5 are important recognition epitopes for CdiA^EC536^. Loops L4 and L5 also account for much of the sequence variability between *E*. *coli* OmpC proteins, raising the possibility that CDI contributes to the selective pressure driving OmpC diversification. We find that the most efficient CdiA^EC536^ receptors are encoded by isolates that carry the same *cdi* gene cluster as *E*. *coli* 536. Thus, it appears that CdiA effectors often bind preferentially to "self" receptors, thereby promoting interactions between sibling cells. As a consequence, these effector proteins cannot recognize nor suppress the growth of many potential competitors. These findings suggest that self-recognition and kin selection are important functions of CDI.

## Author Summary

Bacterial pathogens often live in crowded communities where cells reside in close contact with one another. Many of these bacteria possess contact-dependent growth inhibition (CDI) systems, which allow cells to touch and inhibit each other using toxic CdiA proteins. CDI^+^ bacteria also produce immunity proteins that specifically protect the cell from the CdiA toxins of neighboring sibling cells. The CDI system from *Escherichia coli* EC93 was the first to be characterized and its CdiA toxin recognizes a receptor (BamA) that is identical in virtually all *E*. *coli* isolates. Here, we describe a different CDI system from uropathogenic *E*. *coli* 536, which causes urinary tract infections. In contrast to *E*. *coli* EC93, CdiA from *E*. *coli* 536 binds to receptor proteins (OmpC/OmpF) that vary widely between different *E*. *coli* isolates. Thus, uropathogenic *E*. *coli* preferentially bind and deliver toxins into sibling cells and other closely related *E*. *coli* strains. These results suggest that CDI systems distinguish between "self" and "non-self" cells. Moreover, because sibling cells are immune to CdiA-mediated growth inhibition, these findings raise the possibility that toxin exchange may be used for communication and cooperative behavior between genetically identical bacteria.

## Introduction

Contact-dependent growth inhibition (CDI) systems mediate the transfer of protein toxins between Gram-negative bacteria. CDI was first discovered and characterized in *Escherichia coli* EC93, which uses CdiB^EC93^/CdiA^EC93^ two-partner secretion proteins to inhibit the growth of other *E*. *coli* isolates [1]. CdiB^EC93^ is an Omp85 family β-barrel protein that exports and presents CdiA^EC93^ on the cell surface. CdiA^EC93^ carries toxic effector activity and is predicted to form a β-helical filament that projects several hundred angstroms from the inhibitor cell [2, 3]. CdiA^EC93^ binds to BamA on the surface of neighboring *E*. *coli* cells, then delivers its C-terminal toxin domain (CdiA-CT^EC93^) into the target cell to inhibit growth [4]. The proton gradient is rapidly dissipated in intoxicated cells [5, 6], suggesting that the CdiA-CT^EC93^ toxin forms pores in the cytoplasmic membranes of target bacteria. Because *E*. *coli* EC93 cells also express BamA receptors, they receive toxin domains from neighboring siblings. However, inter-sibling toxin exchange does not induce growth arrest because the *cdi* locus encodes the CdiI^EC93^ immunity protein, which neutralizes CdiA-CT^EC93^ toxicity [1, 7]. Thus, *E*. *coli* EC93 deploys CDI to inhibit competing bacteria, but sibling cells are immune to growth inhibition. Since their discovery in *E*. *coli* EC93, *cdi* genes have been identified and characterized in several other proteobacteria [8–13]. CDI systems are typically encoded on plasmids and genomic islands and therefore are not necessarily found in all isolates of given species. This is exemplified by *E*. *coli*, for which ~25% of sequenced isolates carry *cdi* gene clusters. By contrast, every sequenced strain of *Neisseria meningitidis* and *Burkholderia pseudomallei* encodes at least one CDI system. CDI is also characterized by toxin diversity [3, 14]. CdiA-CT sequences vary widely between bacteria, with toxins exhibiting several distinct activities [3, 15]. CdiI immunity proteins are also diverse and specifically protect against cognate CdiA-CT toxins. Thus, *cdi* genes collectively encode a network of toxin/immunity protein pairs that appear to be rapidly diversifying. Toxin diversity and the specificity of immunity protection suggest that CDI systems are deployed to compete for environmental resources and growth niches.

The molecular mechanisms of CDI toxin delivery have been explored largely using the *E*. *coli* EC93 system as a model. Early work showed that CDI^EC93^ expression in *E*. *coli* K-12 strains promotes auto-adhesion [1] and that CdiA-CT^EC93^ delivery is receptor-dependent [4]. The CdiA^EC93^ receptor, BamA, was identified from selections for CDI-resistant (CDI^R^) target cells. *E*. *coli bamA101* mutants carry a transposon insertion that reduces BamA expression five-fold and confers partial resistance to CDI^EC93^-mediated growth inhibition [4]. BamA is an essential outer-membrane protein that forms the core of the β-barrel assembly machine (BAM) complex in Gram-negative bacteria [16–18]. The BAM complex inserts β-barrel proteins into the outer membrane, and BamA orthologues are found in all Gram-negative bacteria and eukaryotic plastids. Though BamA is conserved, its extracellular loops vary considerably between closely related enterobacterial species [19]. For example, *E*. *coli* BamA^Eco^ shares ~94% overall sequence identity with BamA^ECL^ from *Enterobacter cloacae* ATCC 13047, but the surface-exposed portions of extracellular loops L4, L6 and L7 are unrelated in sequence [20]. Consequently, *E*. *coli* EC93 does not recognize *E*. *cloacae* cells as targets and cannot inhibit the growth of this species. Similarly, replacement of *bamA* ^Eco^ with *bamA* genes from other enterobacterial species confers CDI-resistance to *E*. *coli* cells [20]. Thus, receptor polymorphism restricts the CDI^EC93^ target-cell range to *E*. *coli* and *Shigella* isolates, which share identical BamA proteins. Recognition of "self" receptors appears to be a general feature of CDI, because CdiA effectors from *Dickeya dadantii* 3937, *Burkholderia thailandensis* E264, *Neisseria meningitidis* B16B6 and *Pseudomonas aeruginosa* PAO1 all deliver toxins to sibling cells [8–13]. This exchange of toxins provides no apparent competitive advantage because siblings are immune. However, the accompanying cell-cell adhesion likely imparts significant benefits by promoting auto-aggregation and biofilm formation [7, 11, 13, 21–25]. Thus, in addition to suppressing the growth of competing bacteria, CDI also contributes to bacterial fitness by mediating cooperative behaviors between sibling cells.

Bacterial surfaces interact directly with the environment and are therefore under strong selective pressure. Bacteriophages and adaptive immune systems exploit surface epitopes to identify bacteria for infection and elimination, respectively. In response, bacteria have evolved mechanisms to rapidly alter their repertoire of surface molecules such that different isolates of the same species commonly express unique extracellular antigens [26–29]. Therefore, CdiA effectors from different bacterial species must presumably recognize distinct receptors. Here, we begin to explore CDI receptor diversity using the CdiA^EC536^ effector from uropathogenic *E*. *coli* 536 (EC536). We find that *E*. *coli ompC* and *ompF* mutants are resistant to CDI^EC536^-mediated growth inhibition, suggesting that the OmpC and OmpF osmoporins function as receptors for CdiA^EC536^. CDI^EC536^ inhibitor cells only bind to target bacteria that express OmpC and OmpF, indicating that both porins are required for receptor function. Using single-chain OmpF dimers, we provide evidence that heterotrimeric OmpC-OmpF constitutes the receptor for CdiA^EC536^. Because OmpF and OmpC exhibit considerable sequence variability in their extracellular loops [30, 31], we examined the effects of these polymorphisms on receptor function. We find that several OmpC proteins from uropathogenic *E*. *coli* isolates are not recognized by CdiA^EC536^, suggesting that many potential competitors are resistant to CDI^EC536^. By contrast, OmpC proteins from *E*. *coli* UTI89 and F11 are effective receptors for CdiA^EC536^. Notably, *E*. *coli* UTI89 and F11 also encode CdiA proteins that are >98% identical to CdiA^EC536^, indicating that their effectors also recognize OmpC-OmpF receptors and mediate self-recognition. In these instances, CdiA effector proteins preferentially recognize "self" receptors, suggesting that auto-adhesion and kin selection are important functions of CDI.

## Results

### Genetic analysis of the CDI^EC536^ system

**Fig 1:**
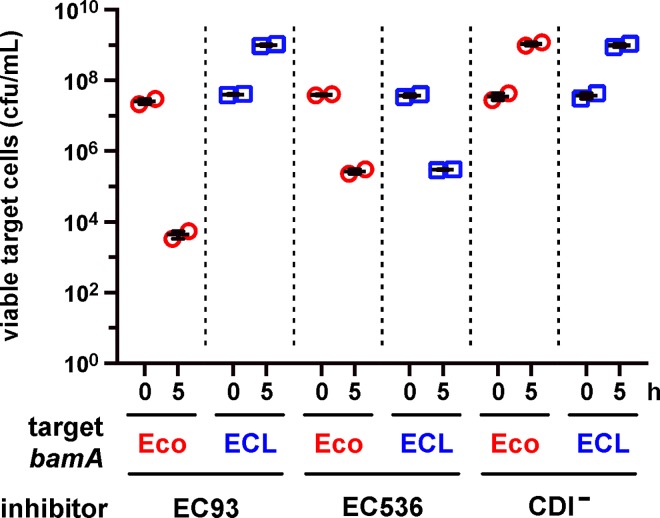
Heterologous BamA does not protect against CDI^EC536^. Target bacteria that express *bamA* ^Eco^ (CH10226) or *bamA* ^ECL^ (CH10227) were cultured at a 1:10 ratio with *E*. *coli* EPI100 inhibitor cells that carry cosmids pDAL660Δ1–39 (CDI^EC93^), pDAL866 (CDI^EC536^) or pWEB::TNC (CDI^–^). Viable target bacteria are reported as colony-forming units per milliliter (cfu/mL) ± SEM for two independent experiments.

We previously reported that *E*. *coli bamA101* mutants are resistant to CDI^EC536^ [32], though the level of protection is less than that observed against CDI^EC93^ inhibitors [4]. Because the *bamA101* mutation confers incomplete resistance, we revisited these experiments using *E*. *coli* target strains that express *E*. *cloacae bamA* (*bamA* ^ECL^), which provides full resistance to CDI^EC93^ [20]. We first confirmed that *E*. *coli bamA* ^ECL^ target cells are resistant to CDI^EC93^ using *E*. *coli* EPI100 inhibitor cells that express the *cdiBAI* ^EC93^ gene cluster from a cosmid vector. As expected, *E*. *coli bamA* ^ECL^ targets grew to the same level in co-culture with either CDI^EC93^ inhibitors or CDI^−^cells that carry an empty cosmid vector (Fig 1). By contrast, *bamA* ^Eco^ target-cell viability decreased ~600-fold during co-culture with CDI^EC93^ inhibitors (Fig 1). We then tested *bamA* ^Eco^ and *bamA* ^ECL^ cells in competitions against *E*. *coli* EPI100 that express the *E*. *coli* 536 *cdiBAI* ^EC536^ genes from a cosmid and found that both target strains were inhibited to the same extent (Fig 1). These results indicate that BamA^ECL^ does not protect against CDI^EC536^, suggesting that the originally observed *bamA101* resistance may be due to decreased expression of an unidentified outer-membrane receptor that requires BamA for assembly.

**Fig 2:**
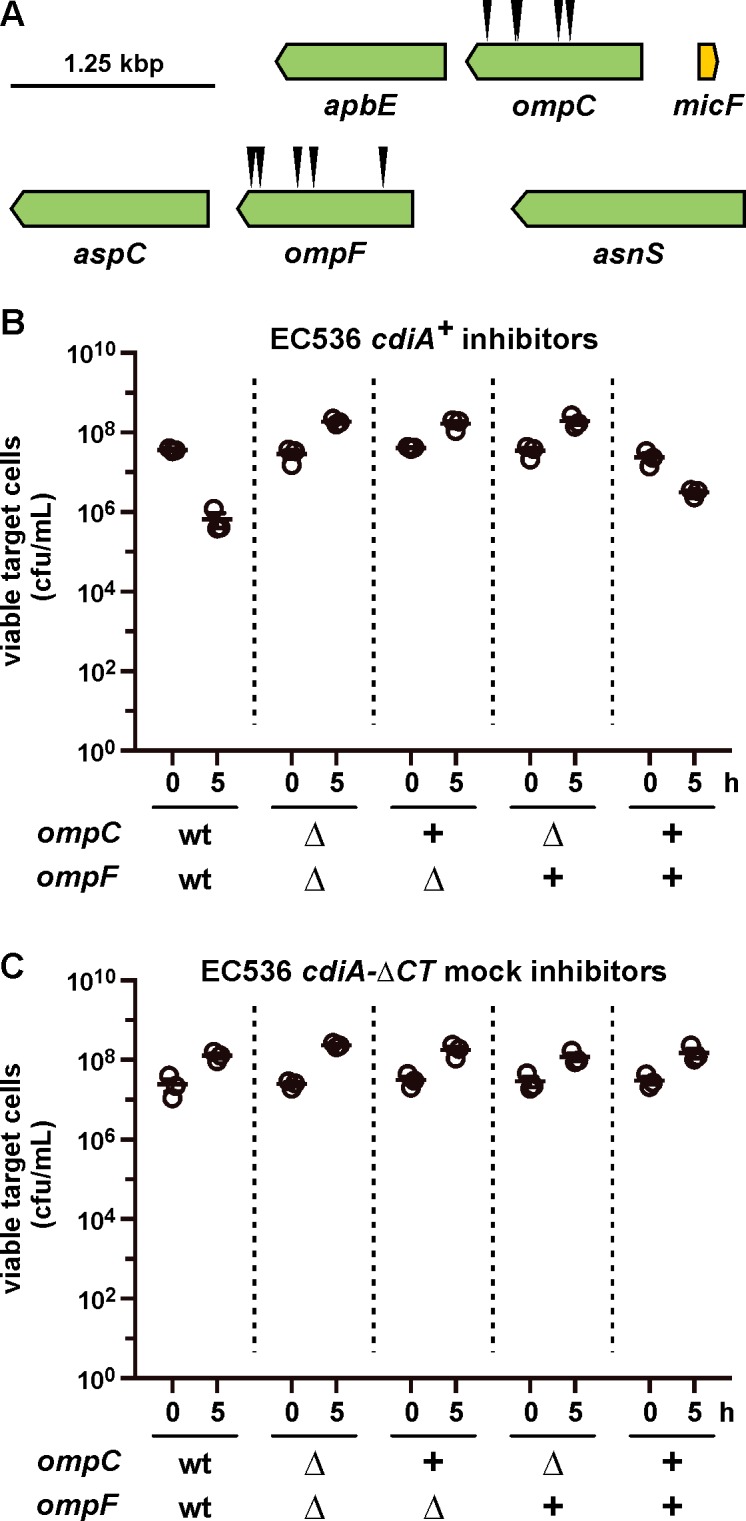
*E*. *coli* Δ*ompC* and Δ*ompF* mutants are resistant to CDI^EC536^. (A) Positions of *mariner* transposon insertion sites from CDI^R^ mutants. (B) Competition co-cultures with CDI^+^ *E*. *coli* 536 inhibitors. *E*. *coli* CH10013 (*ompC* ^*+*^ *ompF* ^*+*^) and CH10136 (Δ*ompC* Δ*ompF*) target cells were co-cultured at a 1:10 ratio with CDI-inducible *E*. *coli* 536 cells. The plus (+) symbols indicate complementation of Δ*ompC* Δ*ompF* target cells with plasmid-borne *ompC* ^K-12^ (pCH10138) and *ompF* ^K-12^ (pCH10202). Viable target bacteria are reported as colony-forming units per milliliter (cfu/mL) ± SEM for three independent experiments. (C) Competition co-cultures with CDI^−^ *E*. *coli* 536. Co-cultures were performed as in panel B, except *E*. *coli* 536 cells deleted for the *cdiA-CT* region were used as mock inhibitors.

We used a genetic approach to identify the CdiA^EC536^ receptor, reasoning that its disruption should confer CDI resistance (CDI^R^). We subjected CDI-sensitive *E*. *coli* target cells to transposon mutagenesis, then selected CDI^R^ mutants from co-cultures with *E*. *coli* 536 cells that express the *cdi* locus under control of an arabinose-inducible promoter. Additionally, because CysK (*O*-acetylserine sulfhydrylase A) is required to activate the CdiA-CT^EC536^ toxin domain [33, 34], we provided target cells with plasmid-borne *cysK* to avoid isolating CDI^R^ *cysK* null mutants. CDI^R^ populations were enriched after three rounds of selection, and randomly isolated clones exhibited complete resistance to growth inhibition. Transposon insertions from CDI^R^ clones were transduced into CDI-sensitive cells and genetic linkage between insertions and CDI^R^ phenotypes was established for each transductant. The disrupted genes were then identified from ten randomly selected CDI^R^ mutants, revealing five independent insertions each in *ompC* and *ompF* (Fig 2A). The *ompC* and *ompF* genes encode closely related osmoporins, which are trimeric β-barrel proteins that facilitate the diffusion of small hydrophilic molecules across the outer membranes of Gram-negative bacteria [35]. To confirm the roles of OmpC and OmpF in CDI^EC536^, we generated an *E*. *coli* Δ*ompC* Δ*ompF* double mutant and performed complementation analysis. As expected, the growth of Δ*ompC* Δ*ompF* cells was not inhibited in co-culture with *E*. *coli* 536 (Fig 2B). By contrast, wild-type *ompC* ^*+*^ *ompF* ^*+*^ target cells showed a 50-fold loss in viability under the same co-culture conditions (Fig 2B). This latter growth inhibition is due to CDI^EC536^, because the growth of wild-type *E*. *coli* was not affected by *E*. *coli* 536 mock inhibitors that carry a deletion of the *cdiA-CT* coding region (Fig 2C). Complementation of the Δ*ompC* Δ*ompF* mutant with a plasmid expressing either *ompC* or *ompF* had no effect on the resistance phenotype, but the strain became CDI-sensitive when it harbored plasmids expressing both osmoporins (Fig 2B). Together, these findings demonstrate that OmpC and OmpF expression is required to render bacteria sensitive to the CDI^EC536^ system.

### OmpC and OmpF are required for CdiA^EC536^-mediated cell-cell adhesion

**Fig 3:**
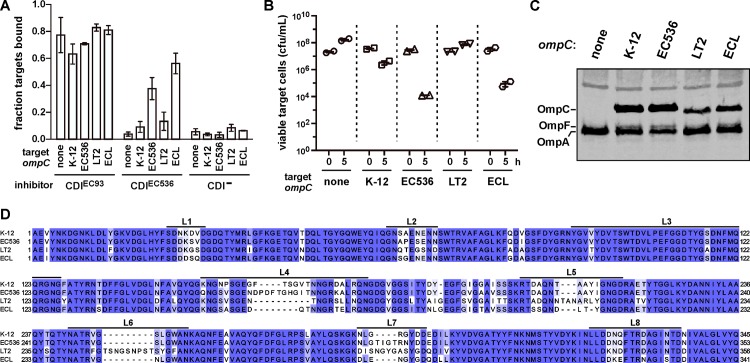
OmpC is required for CDI^EC536^ dependent cell-cell adhesion. (A) Analysis of cell-cell adhesion by flow cytometry. *E*. *coli* DL4905 (GFP^+^) inhibitors carrying cosmids pDAL660Δ1–39 (CDI^EC93^), pDAL866 (CDI^EC536^) or pWEB::TNC (CDI^–^) were mixed at a 5:1 ratio with DsRed-labeled CH10099 (Δ*ompC*) target cells. Where indicated, the target cells were complemented with plasmid-borne *ompC* alleles from *E*. *coli* K-12, *E*. *coli* 536 (EC536), *S*. Typhimurium LT2 or *E*. *cloacae* (ECL). The suspensions were analyzed by flow cytometry using FL1 (533/30nm, GFP) and FL2 (585/40nm, DsRed) fluorophore filters. The percentage of bound target cells was calculated as the number of dual green/red fluorescent events divided by the total number of red fluorescent events. The average ± SEM is presented for two independent experiments. (B) Competition co-cultures with CDI^+^ *E*. *coli* 536. *E*. *coli* CH10099 (Δ*ompC*) target cells were co-cultured at a 1:10 ratio with CDI-inducible *E*. *coli* 536 cells. Where indicated, the target cells were complemented with plasmid-borne *ompC* alleles from *E*. *coli* K-12, *E*. *coli* 536 (EC536), *S*. Typhimurium LT2 or *E*. *cloacae* (ECL). Viable target bacteria are reported as colony-forming units per milliliter (cfu/mL) ± SEM for two independent experiments. (C) Protein from the target bacteria in panel A was subjected to immunoblot analysis using polyclonal antisera against OmpC/F from *E*. *coli* K-12. (D) Sequence alignment of OmpC proteins from *E*. *coli* K-12, *E*. *coli* 536, *S*. Typhimurium LT2 and *E*. *cloacae* ATCC 13047 (ECL). Extracellular loop sequences are indicated above the alignment.

CdiA-mediated adhesion between inhibitor and target cells is a hallmark of CDI. To determine whether OmpC and OmpF are required for CDI-dependent adhesion, we monitored cell-cell binding using a flow-cytometry approach. GFP-labeled inhibitor cells were mixed with DsRed-labeled target bacteria at a 5:1 ratio, and the suspensions analyzed by flow cytometry. Flow events registering both green and red fluorescence correspond to aggregates containing inhibitor and target bacteria. The flow cytometric assay for CDI-mediated adhesion generally requires CdiA over-expression [4, 36], therefore we used inhibitor cells that express *cdi* genes from cosmids for these experiments. Under these conditions, CDI^EC93^ inhibitor cells bind to 60–80% of target bacteria (Fig 3A). This cell-cell adhesion is dependent on CDI, with only about 5% of target cells associated with CDI^−^mock inhibitors (Fig 3A). We then tested whether CDI^EC536^ expression also promotes stable adhesion to target cells. As expected, we did not detect CDI^EC536^-dependent adhesion to Δ*ompC* target bacteria; but surprisingly there was little increase in adhesion upon complementation with *ompC* ^K-12^ from *E*. *coli* K-12 (Fig 3A). Despite the lack of detectable cell-cell adhesion, *ompC* ^K-12^-complemented cells were inhibited during co-culture with *E*. *coli* 536 cells (Fig 3B) and immunoblotting confirmed that they produced OmpC^K-12^ (Fig 3C). OmpC porins vary significantly between *E*. *coli* isolates [30], and the *E*. *coli* K-12 and 536 proteins carry different surface-exposed loops (Fig 3D). Because extracellular loop polymorphism could affect receptor function, we tested whether *ompC* ^EC536^ better supports CDI^EC536^-dependent cell adhesion. Strikingly, over 40% of *ompC* ^EC536^ complemented bacteria adhered to CDI^EC536^ inhibitors, and this adhesion was specific because these target cells did not associate with CDI^−^mock inhibitors (Fig 3A). These results suggest that CdiA^EC536^ binds to OmpC^EC536^ with higher avidity than OmpC^K-12^. We also found that *ompC* ^EC536^ target cells were inhibited to a greater extent than *ompC* ^K-12^ targets in co-cultures with *E*. *coli* 536 (Fig 3B). We next tested OmpC proteins from *Salmonella* Typhimurium LT2 and *E*. *cloacae* ATCC 13047 (Fig 3D) to determine whether other species are potential targets for the CDI^EC536^ system. *E*. *coli* Δ*ompC* cells complemented with *S*. Typhimurium *ompC* ^LT2^ did not adhere to CDI^EC536^ inhibitors (Fig 3A) and were not inhibited in competition co-culture (Fig 3B), though they produced substantial OmpC^LT2^ protein (Fig 3C). By contrast, CDI^EC536^ inhibitors bound to *ompC* ^ECL^ complemented cells and inhibited their growth (Fig 3A and 3B). Together, these results support a role for OmpC as the CdiA^EC536^ receptor and indicate that OmpC polymorphism determines whether bacteria are susceptible to CDI^EC536^-mediated growth inhibition.

**Fig 4:**
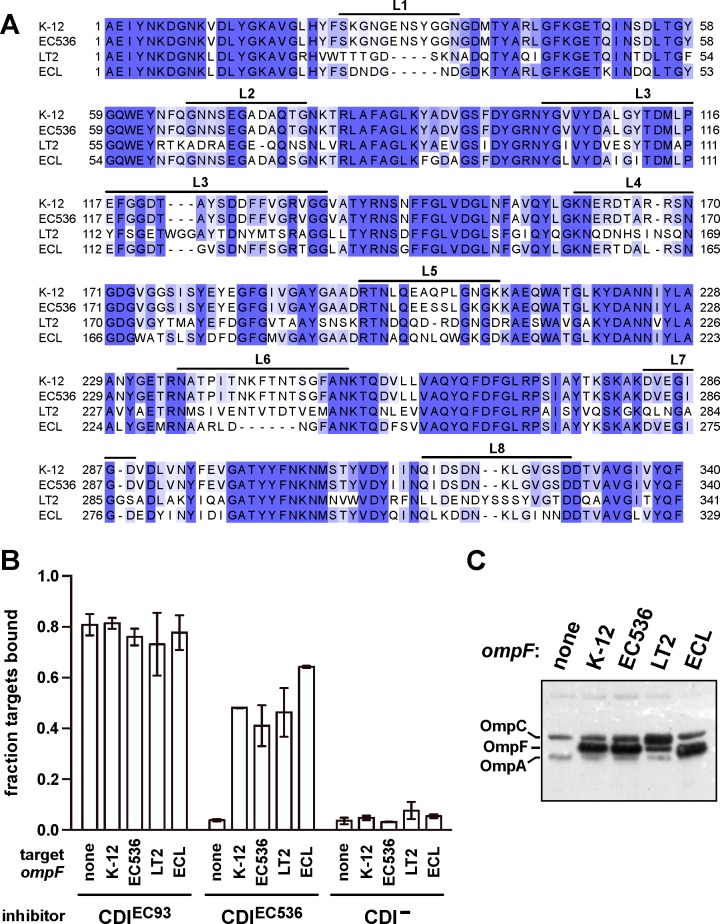
OmpF is required for CDI^EC536^ dependent cell-cell adhesion. (A) Sequence alignment of OmpF proteins from *E*. *coli* K-12, *E*. *coli* 536, *S*. Typhimurium LT2 and *E*. *cloacae* ATCC 13047 (ECL). Extracellular loop sequences are indicated above the alignment. (B) Analysis of cell-cell adhesion by flow cytometry. *E*. *coli* DL4905 (GFP^+^) inhibitors carrying cosmids pDAL660Δ1–39 (CDI^EC93^), pDAL866 (CDI^EC536^) or pWEB::TNC (CDI^–^) were mixed at a 5:1 ratio with DsRed-labeled CH12134 (Δ*ompF ompC* ^EC536^) target cells. Where indicated, target cells were complemented with plasmid-borne *ompF* from *E*. *coli* K-12, *E*. *coli* 536 (EC536), *S*. Typhimurium LT2 or *E*. *cloacae* (ECL). Cells suspensions were analyzed by flow cytometry using FL1 (533/30nm, GFP) and FL2 (585/40nm, DsRed) fluorophore filters. The percentage of bound target cells was calculated as the number of dual green/red fluorescent events divided by the total number of red fluorescent events. The average ± SEM is presented for two independent experiments. (C) Protein from the target bacteria in panel B was subjected to immunoblot analysis using polyclonal antisera against OmpC/F from *E*. *coli* K-12.

We next examined the contribution of OmpF to CDI^EC536^-dependent adhesion by testing whether porins from different bacteria influence receptor activity (Fig 4A). Because OmpC^K-12^ does not support detectable adhesion to CDI^EC536^ inhibitor cells, we replaced the native *ompC* ^K-12^ locus with the *ompC* ^EC536^ allele to generate a target-cell strain for cell-cell binding studies. The resulting Δ*ompF ompC* ^EC536^ cells did not form aggregates with CDI^EC536^ inhibitors, but adhered normally to CDI^EC93^ inhibitor cells (Fig 4B). We then expressed plasmid-borne *ompF* from *E*. *coli* K-12, *E*. *coli* 536, *S*. Typhimurium and *E*. *cloacae* and found that each allele supported adhesion to CDI^EC536^ inhibitors (Fig 4B and 4C). These results demonstrate that both OmpC and OmpF are required for CDI^EC536^-dependent cell adhesion, indicating that each porin contributes to receptor function.

### CdiA^EC536^ recognizes OmpC-OmpF heterotrimers

**Fig 5:**
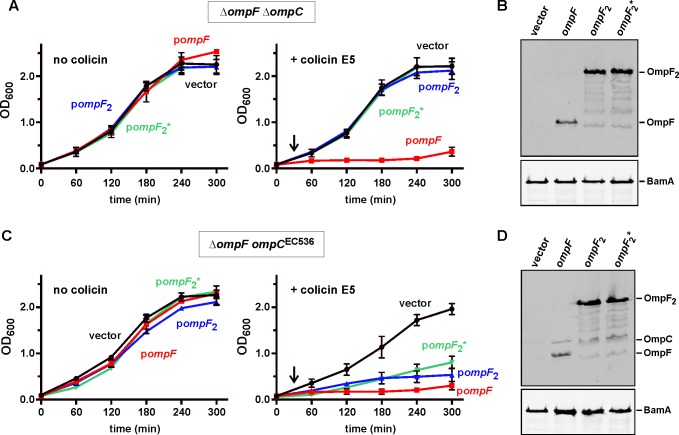
Characterization of functional OmpF-OmpC heterotrimers. (A) *E*. *coli* CH11346 (Δ*ompF ΔompC*) cells were complemented with *ompF* (pCH11850), *ompF-ompF* with 8-residue linker (***ompF***_**2**_, pCH10796), or *ompF-ompF* with 16-residue linker (***ompF***_**2**_ *, pCH12054) and the resulting strains cultured in LB media without or with colicin E5 (added at 30 min as indicated by the arrow). Cell growth was monitored by measuring the optical density at 600 nm (OD_600_) of the cultures. Data are from two independent experiments. (B) Protein from the strains in panel A was subjected to immunoblot analysis using polyclonal antisera against OmpC/F and BamA (as a loading control) from *E*. *coli* K-12. (C) *E*. *coli* CH12180 (Δ*ompF ompC* ^EC536^) cells were complemented with plasmid-borne *ompF*, *ompF*_2_ or *ompF*_2_* and cultured as described in panel A. (D) Protein from the strains in panel C was subjected to immunoblot analysis using polyclonal antisera against OmpC/F from *E*. *coli* K-12.

**Fig 6:**
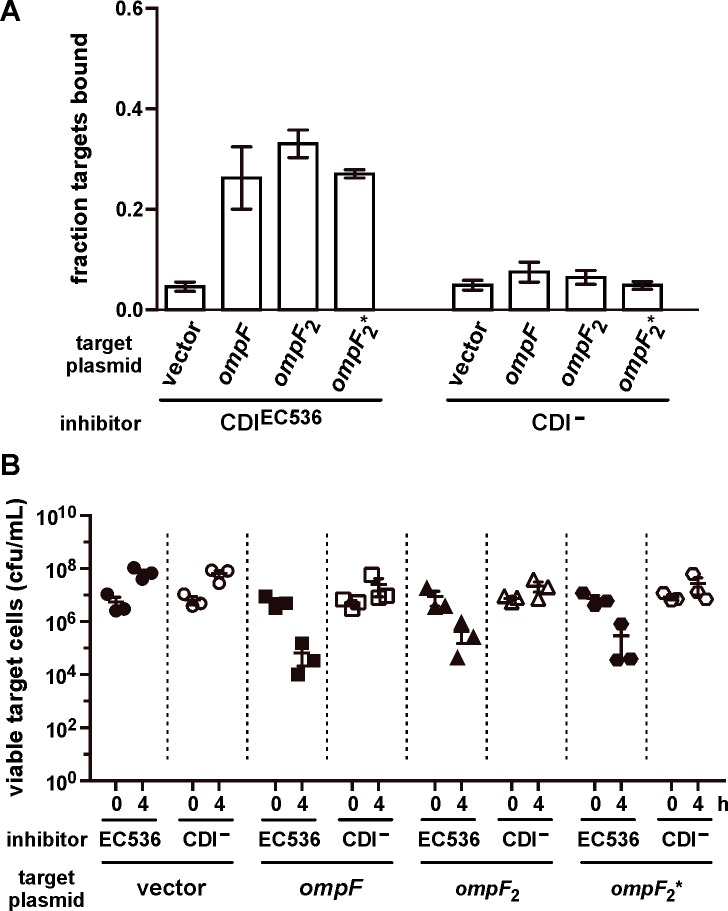
Heterotrimeric OmpF-OmpC supports CDI^EC536^-mediated growth inhibition. (A) *E*. *coli* DL4905 (GFP^+^) inhibitors carrying pDAL866 (CDI^EC536^) or pWEB::TNC (CDI^–^) were mixed at a 5:1 ratio with DsRed-labeled *E*. *coli* CH12180 (Δ*ompF ompC* ^EC536^) target cells complemented with *ompF* (pCH11850), *ompF-ompF* with 8-residue linker (***ompF***_**2**_, pCH10796), or *ompF-ompF* with 16-residue linker (***ompF***_**2**_ *, pCH12054). Cell suspensions were analyzed by flow cytometry using FL1 (533/30nm, GFP) and FL2 (585/40nm, DsRed) fluorophore filters. The fraction of bound target cells was calculated as the number of dual green/red fluorescent events divided by the total number of red fluorescent events. The average ± SEM is presented for two independent experiments. (B) *E*. *coli* CH12180 (Δ*ompF ompC* ^EC536^) target cells were complemented were complemented with plasmid-borne *ompF*, *ompF*_2_ or *ompF*_2_* and the resulting strains co-cultured at a 1:10 ratio with CDI^+^ (filled symbols) or CDI^−^(open symbols) *E*. *coli* 536 cells. Viable target bacteria are reported as colony-forming units per milliliter (cfu/mL) ± SEM for three independent experiments.

At least two models can explain the roles of OmpC and OmpF in CDI. CdiA^EC536^ could interact simultaneously with adjacent OmpC and OmpF homotrimers on the target-cell surface. Alternatively, the effector could recognize OmpC and OmpF displayed as heterotrimers, which have been reported previously [37]. To differentiate between these models, we generated and tested the function of covalently constrained OmpC-OmpF heterotrimers. Because osmoporins are obligate trimers, we reasoned that porin dimers produced from a gene fusion should require an additional monomer to assemble properly in the outer membrane. We tested this prediction using dimeric OmpF proteins tethered by 8- and 16-residue linker peptides. OmpF assembly and function was assessed by monitoring sensitivity to colicin E5, which requires OmpF to translocate its C-terminal toxin domain into *E*. *coli* cells [38–41]. *E*. *coli* Δ*ompF* Δ*ompC* mutants are completely resistant to colicin E5, but become susceptible when complemented with plasmid-borne *ompF* ^EC536^ (Fig 5A). By contrast, neither of the dimeric *ompF* constructs restored colicin sensitivity (Fig 5A), even though immunoblot analysis confirmed the presence of OmpF dimers in these cells (Fig 5B). We then expressed *ompF-ompF* fusions in the Δ*ompF ompC* ^EC536^ background to test whether monomeric OmpC supports heterotrimer formation. *E*. *coli* Δ*ompF ompC* ^EC536^ cells are somewhat sensitive to colicin E5 (Fig 5C), consistent with a prior report showing that OmpC can support colicin entry in the absence of OmpF [42]. However, colicin E5 had a greater inhibitory effect on Δ*ompF ompC* ^EC536^ cells that were complemented with plasmids that produce either monomeric or dimeric OmpF (Fig 5C). Together, these results indicate that OmpF_2_-OmpC heterotrimers assemble in the outer membrane and are active in colicin translocation. Having established a strategy to generate heterotrimeric porin complexes, we tested whether OmpF_2_-OmpC is a receptor for CdiA^EC536^. Flow cytometry showed that each OmpF dimer supported CDI^EC536^-dependent cell-cell adhesion to the same extent as monomeric OmpF (Fig 6A). Moreover, we also found that Δ*ompF ompC* ^EC536^ cells expressing dimeric OmpF were inhibited 10- to 100-fold by *E*. *coli* 536 cells in competition co-cultures (Fig 6B). Taken together, these results indicate that heterotrimeric OmpF-OmpC is the receptor for CdiA^EC536^.

### OmpC loops L4 and L5 are recognition determinants for CdiA^EC536^

**Fig 7:**
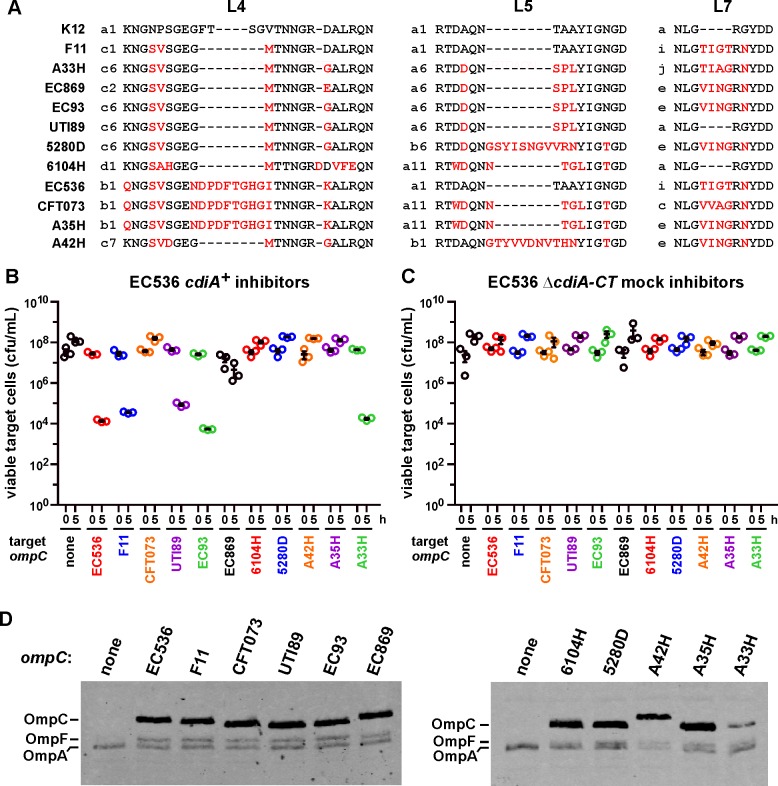
*E*. *coli* OmpC polymorphism restricts the target-cell range of CDI^EC536^. (A) Extracellular loop L4, L5 and L7 sequences from selected *E*. *coli* isolates. S1 Fig shows the complete sequence alignment, and the alphanumeric classification scheme for L4, L5 and L7 sequences is presented in S2 Table. (B) *E*. *coli* CH10099 (Δ*ompC*) target cells were co-cultured at a 1:10 ratio with CDI^+^ *E*. *coli* 536 cells. Target cells were complemented with plasmid-borne *ompC* alleles from the indicated *E*. *coli* isolates. Viable target bacteria are reported as colony-forming units per milliliter (cfu/mL) ± SEM for three independent experiments. (C) The same target-cell strains from panel B were co-cultured at a 1:10 ratio with CDI^−^ *E*. *coli* 536 and viable cells quantified in the same manner. (D) Protein from *ompC* complemented strains was subjected to immunoblot analysis using polyclonal antisera against OmpC/F from *E*. *coli* K-12.

We next used natural variations between *E*. *coli* OmpC porins to identify potential CdiA^EC536^ recognition epitopes. We surveyed predicted OmpC proteins from *E*. *coli* isolates and identified over 220 distinct sequences (S1 Table). Though some *E*. *coli* OmpC variants differ by single amino-acid residue substitutions, much of the sequence variation is localized to extracellular loops L4 (33 sequences), L5 (30 sequences) and L7 (13 sequences) (S2 Table). Moreover, these loops are found in many different combinations (S1 Table). To examine the influence of OmpC polymorphism on CDI^EC536^, we tested a subset of *E*. *coli* OmpC proteins with unique extracellular loop combinations (Figs 7A and S1). *E*. *coli* Δ*ompC* target cells were provided with plasmid-borne *ompC* alleles and co-cultured with *E*. *coli* 536 inhibitor cells. The *ompC* alleles from *E*. *coli* UTI89, F11 and A33H rendered Δ*ompC* cells sensitive to growth inhibition to roughly the same extent as *ompC* ^EC536^ (Fig 7B). Targets that express *ompC* ^EC93^ from *E*. *coli* EC93 were also inhibited in co-culture with *E*. *coli* 536, but *ompC* alleles from uropathogenic isolates CFT073, 6104H, A54H, A42H and A35H were all associated with resistance (Fig 7B and 7C). Immunoblot analysis confirmed that OmpC was produced in all of the complemented strains (Fig 7D), indicating that resistance is not due to a lack of *ompC* expression. Thus, *E*. *coli ompC* allele polymorphism appears to dictate cell susceptibility to CDI^EC536^-mediated intoxication.

**Fig 8:**
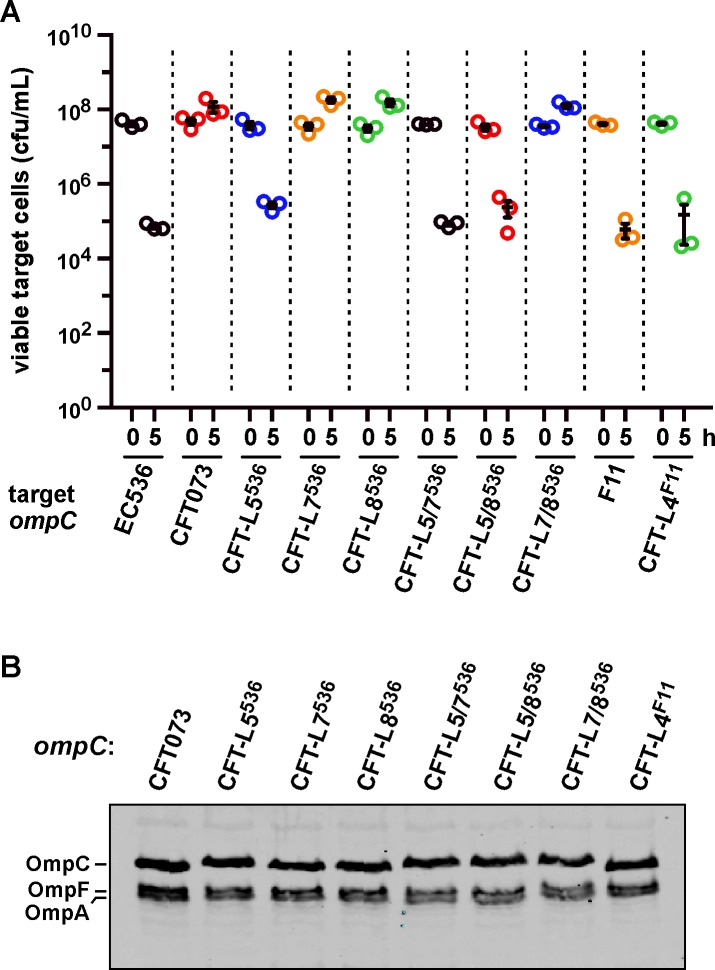
OmpC extracellular loops L4 and L5 are important recognition determinants for CdiA^EC536^. (A) *E*. *coli* CH10099 (Δ*ompC*) target cells were co-cultured at a 1:10 ratio with CDI^+^ *E*. *coli* 536 cells. Target cells were complemented with the indicated chimeric *ompC* alleles. Viable target bacteria are reported as colony-forming units per milliliter (cfu/mL) ± SEM for three independent experiments. (B) Protein from the strains in panel A was subjected to immunoblot analysis using polyclonal antisera against OmpC/F from *E*. *coli* K-12.

To determine which OmpC sequences are critical for recognition by CdiA^EC536^, we generated chimeric porins using OmpC^CFT073^ as a framework upon which to graft heterologous OmpC loops. OmpC^CFT073^ and OmpC^EC536^ share loop L4, but differ in their L5, L7 and L8 sequences (Figs 7A and S1). OmpC^CFT073^ chimeras carrying L7 or L8 from OmpC^EC536^ still provided resistance to CDI^EC536^ (Fig 8A), indicating that these loops probably have no role in binding CdiA^EC536^. However, replacement of loop L5 converted OmpC^CFT073^ into a functional receptor that supports the same level of inhibition as OmpC^EC536^ (Fig 8A). Similarly, OmpC^CFT073^ chimeras carrying OmpC^EC536^ L7 or L8 were functional receptors, provided that they also carried loop L5 from OmpC^EC536^ (Fig 8A). We tested the role of loop L4 by grafting the sequence from OmpC^F11^ (which is a receptor for CdiA^EC536^) onto OmpC^CFT073^. Targets that express the resulting chimera were inhibited by *E*. *coli* 536 to about the same extent as targets that express OmpC^F11^ (Fig 8A). This latter observation shows that OmpC^CFT073^ loop L5 is not an anti-determinant, because it supports CdiA^EC536^ recognition in other contexts. Collectively, these results indicate that OmpC loops L4 and L5 contribute to target-cell selection, suggesting that the loops may be recognized directly by CdiA^EC536^.

## Discussion

The results presented here establish a critical role for OmpC and OmpF in the CDI^EC536^ pathway. CDI^EC536^ inhibitor cells only adhere to target bacteria that express OmpC and OmpF, indicating that both porins contribute to receptor function. Though osmoporins are generally assumed to form homotrimers, we show that heterotrimeric OmpC-OmpF constitutes the CdiA^EC536^ receptor. Heterotrimeric porins were first reported by Gehring and Nikaido, who found that OmpC, OmpF and PhoE can form mixed trimers in *E*. *coli* K-12 [37]. OmpC^K-12^ and OmpF^K-12^ share 58.5% sequence identity (S2 Fig), and their β-barrels superimpose with root-square mean deviation of 0.78 Å [43]. The trimer interaction surface is dominated by hydrophobic contacts between β-barrel strands β1, β2, β3, β4, β5 and β1´, and these regions are highly conserved between OmpC^K-12^ and OmpF^K-12^ (S2 and S3 Figs, S3 Table). The porins also share an inter-protomer ion-pair between a conserved Glu residue (Glu66 in OmpC^K-12^ and Glu71 in OmpF^K-12^) at the tip of loop L2 and an Arg residue within L3 of the adjacent β-barrel (S3 Fig and S3 Table). Thus, the structures of OmpF and OmpC are compatible with heterotrimer formation. The targeting of heterotrimeric OmpF-OmpC receptors has a number of implications. Firstly, residues from both porins should be directly recognized by CdiA^EC536^. The results presented here suggest that OmpC loops L4 and L5 are important CdiA^EC536^-binding determinants, but the contribution of OmpF is less clear. OmpF porins from *E*. *cloacae* and *S*. Typhimurium are effective co-receptors in conjunction with OmpC^EC536^, so the CdiA-binding epitope is presumably shared by all of the tested OmpF variants. Additionally, because OmpC and OmpF are regulated reciprocally, it is possible that receptor availability fluctuates in response to environmental conditions. OmpF is expressed preferentially in response to low osmolarity, whereas OmpC dominates under hyperosmotic conditions [35]. We find that target cells are effectively inhibited when they over-express *ompC* alleles, suggesting that OmpF-OmpC_2_ heterotrimers are recognized by CdiA^EC536^. We have also shown that covalently constrained OmpF_2_-OmpC heterotrimers are functional receptors. Because both heterotrimeric forms support CDI^EC536^, we conclude that CdiA^EC536^ effectors probably make contact with one OmpC and one OmpF protomer in the receptor complex.

The mechanism(s) by which CDI toxins are translocated across the target-cell outer membrane is unknown, but in principle, receptors could mediate this transport. Group A colicins appear to exploit the central pore of OmpF to cross the outer membrane, and this translocation is powered by the proton-motive force via the Tol protein complex [40, 41, 44, 45]. However, CDI toxin translocation is not Tol-dependent, nor does it require the Ton-Exb system, which energizes the import of group B colicins [6, 44]. Further, CDI toxin domains are modular and can be exchanged between CdiA proteins for delivery through different receptor pathways [8, 10, 46]. Therefore, if CdiA receptors mediate outer-membrane transport, then BamA must also be capable of CDI toxin translocation. Like OmpF and OmpC, BamA is a 16-strand β-barrel protein, but its lumen is not exposed to the extracellular milieu. Instead, extracellular loops L3, L4, L6, L7 and L8 form a dome that shields the BamA lumen from the environment [47, 48]. It is possible that the BamA barrel opens upon binding to CdiA^EC93^, consistent with work showing that extracellular loop L6 is dynamic during the OMP assembly duty cycle [49, 50]. Buchanan and colleagues have also suggested that the BamA β-barrel opens a lateral gate during OMP biogenesis [51]. These conformational changes could be exploited to translocate toxin across the outer membrane, but we note that inactive BamA lacking the POTRA-3 (polypeptide-transport associated) domain is able to complement the *bamA101* mutation and restore sensitivity to CDI^EC93^ [4]. Therefore, OMP biogenesis activity is not required for CDI, and thus it remains unclear whether BamA dynamics contribute to toxin translocation.

Putative CdiA-binding epitopes have been localized on BamA and OmpC, but the corresponding receptor-binding regions within CdiA^EC93^ and CdiA^EC536^ have not yet been identified. Given that BamA and OmpF/OmpC have distinct structures, the two CdiA effectors should contain unique receptor-binding domains. Alignment of CdiA^EC93^ and CdiA^EC536^ shows several regions of sequence divergence. Prominent among these are the C-terminal toxin region and the sequence corresponding to residues Ser1379 –Tyr1636 of CdiA^EC93^ (S4 Fig). Additionally, the CdiA^EC536^ effector contains a 69-residue insertion (Ser1988 –Ser2056) relative to CdiA^EC93^ (S4 Fig). We recently reported that truncated CdiA^EC93^ proteins lacking up to ~1,000 C-terminal residues retain BamA-binding function [7]. Furthermore, CdiA^EC93^ lacking internal residues Ala722 –Gly1624 is still secreted and assembled onto the cell surface, but does not bind to BamA [7]. Together, these observations suggest that the receptor-binding domain lies between residues Ser1300 –Asn1600 of CdiA^EC93^. There are four distinct classes of *E*. *coli* CdiA proteins based on sequence comparisons of the 1300–1600 region, suggesting that there may be additional CDI receptors in *E*. *coli*. Alternatively, the other uncharacterized *E*. *coli* CdiA effectors could bind to BamA or OmpC/OmpF using epitopes that are distinct from those recognized by CdiA^EC93^ and CdiA^EC536^ (respectively). Intriguingly, the putative receptor-binding domain is located in the middle of the CdiA primary sequence, between the FHA-1 and FHA-2 peptide repeat regions (S4 Fig). This position is unexpected based on current models of CdiA structure and cell-surface presentation. According to the β-helix structural model [2], the receptor-binding region would be positioned near the center of the CdiA filament rather than the distal tip. It is unclear how a central location is compatible with receptor binding, because the distal end of filament would presumably have to perforate the target-cell outer membrane. Penetration of target bacteria could facilitate toxin delivery, but this model raises the question of why specific receptors should be required for CDI.

Bacterial surface antigens are under intense pressure to diversify, yet *E*. *coli* BamA and OmpC proteins show remarkably different responses to this selective pressure. BamA is invariant in over 2,500 *E*. *coli* isolates for which sequence information is available. By contrast, *E*. *coli* strains encode at least 220 distinct OmpC proteins, many of which are distinguished by unique combinations of extracellular loops L4, L5 and L7. Crystal structures are available for the OmpC^K-12^, OmpC^CFT073^ and OmpC^6104H^ porins studied here, and each exhibits a distinct surface topology and electrostatic potential (S5 Fig) [43, 52]. The large number of extracellular loop combinations seen in *E*. *coli* OmpC proteins suggests that these elements are modular and can be rearranged independently to generate novel porins. However, OmpC loops L4, L5, L6, L7 and L8 engage in a series of intra-protomer interactions (S4 Table), and therefore some loop combinations may be incompatible with a properly folded porin structure. Most intra-protomer loop interactions (e.g. L5-L7 and L7-L8) are mediated by invariant residues, though the contacts between L4 and L5 in OmpC^CFT073^ and OmpC^6104H^ involve non-conserved residues (S4 Table). We note that chimeric OmpC^CFT073^ carrying L4 from OmpC^F11^ represents a L4-L5 combination that has yet to be found in any *E*. *coli* isolate. Because this OmpC chimera is functional as a CdiA^EC536^ receptor, it must be assembled properly in the outer membrane. Therefore, in this instance, loop L4 is clearly modular. This combinatorial complexity has implications for CDI, and almost certainly governs bacterial susceptibility to bacteriophages and immune recognition [53, 54].

CDI receptor polymorphism restricts the number of susceptible target bacteria, yet CdiA^EC536^ recognizes multiple OmpC variants. OmpC proteins from *E*. *coli* EC93, 536, F11, UTI89 and A33H are all recognized efficiently by CdiA^EC536^ though each receptor carries a distinct combination of extracellular loops. *E*. *coli* F11 and UTI89 encode CdiA proteins that share >98% identity with CdiA^EC536^. Taken together, these observations indicate that the CdiA^F11^ and CdiA^UTI89^ effectors also bind to OmpF-OmpC and preferentially recognize self-receptors. These findings are consistent with numerous reports that CDI^+^ bacteria tend to auto-aggregate [1, 7, 11, 13, 21–23]. Intriguingly, *E*. *coli* CFT073 also encodes a CdiA effector that shares 89.7% identity with CdiA^EC536^ over 3,037 N-terminal residues, yet OmpC^CFT073^ does not function as a CdiA^EC536^ receptor. Inspection of the *cdiB* gene from *E*. *coli* CFT073 reveals that it contains an in-frame stop codon [55], and therefore this strain presumably cannot secrete its CdiA effector. It is tempting to speculate that the loss of CdiA^CFT073^ surface expression has allowed *ompC* ^CFT073^ to diverge in response to other selective pressures. However, there are other *E*. *coli* isolates with intact *cdi* loci that also share *ompC* and *ompF* alleles with CFT073. Based on the data presented here, it appears that the CdiA proteins from these latter isolates would not preferentially recognize self and therefore may function primarily in competition. Though auto-adhesion is beneficial for bacteria, these phenomena can also be viewed from the perspective of *cdi* genes as selfish elements. CDI systems are encoded on mobile genetic elements and their toxin/immunity activities act as "gene-addiction" modules [56]. Cells that lose CDI-encoding plasmids or genomic islands are suddenly susceptible to inhibition and risk elimination by neighboring CDI^+^ siblings. Thus, self-recognition would ensure continual selective pressure to retain *cdi* genes in a population. We speculate that CdiA effectors may have first evolved as adhesins used exclusively for cooperation. Toxin-delivery capabilities could then be grafted onto the ancestral CdiA protein, providing a strong positive selection for *cdi* retention. Thus, the antagonistic activity of CDI systems may have arisen from evolutionary pressure to retain *cdi* genes in fluid bacterial genomes.

## Materials and Methods

### Bacterial strains

Bacterial strains are listed in Table 1. Bacteria were grown in lysogeny broth (LB) or on LB agar unless otherwise noted. Where indicated, media were supplemented with antibiotics at the following concentrations: ampicillin, 150 μg mL^−1^; kanamycin, 50 μg mL^−1^; chloramphenicol, 66 μg mL^−1^; spectinomycin, 50 μg mL^−1^; rifampicin, 200 μg mL^−1^; streptomycin, 100 μg mL^−1^. The *bamA*::*cat* allele was introduced into MC4100 pZS21-*bamA* ^+^ cells by bacteriophage P1-mediated transduction [20]. The *ΔompC*::*kan*, *ΔompF*::*kan*, *Δwzb*::*kan* and *ΔrecA*::*kan* disruptions were obtained from the Keio collection [57] and transduced into *E*. *coli* strain CH10013. Kanamycin-resistance cassettes were subsequently removed with FLP recombinase expressed from plasmid pCP20 [58]. The *ompC* ^K-12^ gene of *E*. *coli* CH10013 was replaced with *ompC* ^EC536^ using Red-mediated recombination. *ompC* ^EC536^ was amplified by PCR with oligonucleotides 3322 and 3323, and a fragment upstream of *ompC* ^K-12^ was amplified using oligonucleotide pair 3320/3321 (all oligonucleotide primers are listed in S5 Table). The two DNA fragments were combined into one fragment by overlap-extension PCR (OE-PCR) using primers 3320/3323 and ligated to pKAN [59] using SacI/BamHI sites to generate plasmid pCH11341. A fragment downstream of *ompC* ^K-12^ was then amplified with primers 3324/3325 and ligated to pKAN using EcoRI/KpnI sites to generate plasmid pCH11342. Fragments from pCH11341 and pCH11342 were amplified with primer pairs 3320/3505 and 3504/3325 (respectively) and the resulting products combined into one fragment by OE-PCR using primers 3320/3325. The final product was introduced into *E*. *coli* CH10013 that express phage λ Red proteins from plasmid pSIM6 [60] and recombinant bacteria selected on LB agar supplemented with kanamycin.

**Table 1:** Bacterial strains.

*Bacterial strain*	*Description* ^a^	*Reference*
EC536	uropathogenic isolate of *E*. *coli*	[66]
CFT073	uropathogenic isolate of *E*. *coli*	[67]
F11	uropathogenic isolate of *E*. *coli*	[68]
UTI89	uropathogenic isolate of *E*. *coli*	[69]
6104H	uropathogenic isolate of *E*. *coli*	[70]
A54H	uropathogenic isolate of *E*. *coli*	[70]
A42H	uropathogenic isolate of *E*. *coli*	[70]
A35H	uropathogenic isolate of *E*. *coli*	[70]
A33H	uropathogenic isolate of *E*. *coli*	[70]
EC93	*E*. *coli* isolate from rat feces	[1]
EPI100	F^−^ *mcrA* Δ*(mrr-hsdRMS-mcrBC) φ80dlacZ*Δ*M15* Δ*lacXcZ*Δ*M15* Δ*lacX recA1 endA1 araD139* Δ*(ara*, *leu)7697 galU galK* λ*^−^rpsL nupG*	Epicentre
MFD*pir*	RP4-2 Tc::[Δ*Mu1*::*aac(3)IV*-Δ*aphA*-Δ*nic35*-Δ*Mu2*::*zeo*] Δ*dapA*::*(erm-pir)* Δ*recA*	[62]
DL4905	MC4100 λ640–13 P_*papBA*_-*gfp-mut3*, Kan^R^	[71]
DL6536	*E*. *coli* 536 Δ*kps15*::*cat* Δ*araBAD spc-araC*-P_*ara*_::*cdiBAI*, Cm^R^ Str^R^ Spc^R^	[8]
DL6381	*E*. *coli* 536 Δ*kps15*::*cat* Δ*araBAD spec-araC*-P_*ara*_::*cdiBA(ΔCT)I*, Cm^R^ Str^R^ Spc^R^	[8]
CH2016	X90 (DE3) Δ*rna* Δ*slyD*::*kan*, Kan^R^	[72]
CH10013	spontaneous rifampicin-resistant isolate of JCM158, Rif^R^	[73]
CH10099	CH10013 Δ*ompC*	This study
CH10100	CH10013 Δ*ompF*	This study
CH10136	CH10013 Δ*ompC* Δ*ompF*	This study
CH10226	MC4100 Δ*bam*::*cat* pZS21*amp*-*bamA* ^Eco^, Amp^R^	This study
CH10227	MC4100 Δ*bam*::*cat* pZS21*amp*-*bamA* ^ECL^, Amp^R^	This study
CH11346	CH10013 Δ*wzb* Δ*ompF* Δ*ompC* Δ*recA*, Rif^R^	This study
CH12133	CH10013 *ompC* ^EC536^ *-kan*, Kan^R^	This study
CH12134	CH10013 Δ*ompF ompC* ^EC536^ *-kan*, Kan^R^	This study
CH12180	CH10013 Δ*wzb* Δ*ompF ompC* ^EC536^ Δ*recA*::*kan*, Rif^R^ Kan^R^	This study
CH12345	JCM158 Δ*wzb* Δ*ompF* Δ*ompC* Δ*recA*::*kan*, Kan^R^	This study

^a^Abbreviations: Amp^R^, ampicillin resistant; Cm^R^, chloramphenicol resistant; Kan^R^, kanamycin resistant; Rif^R^, rifampicin resistant; Spc^R^, spectinomycin resistant; Str^R^, streptomycin resistant

### Plasmid constructions

Plasmids constructs used in this study are listed in Table 2. The *E*. *coli cysK* gene was amplified with primer pair 2111/2110 and ligated to pBR322 using EcoRV and BamHI restriction sites to generate plasmid pCH9763. The *ompF* ^K-12^ allele was amplified from *E*. *coli* JCM158 with primer pair 2385/2386, and the fragment ligated to pZS21 using BamHI and XbaI restriction sites to generate plasmid pCH10202. Additionally, *ompF* ^K-12^ and *ompF* ^EC536^ were amplified with primers 2729/2734, and the fragments ligated to pTrcKX using KpnI and XhoI restriction sites to generate plasmids pCH11850 and pCH10780. The *ompF* genes from *S*. Typhimurium LT2 and *E*. *cloacae* ATCC 13047 were amplified with primer pairs 3468/3469 and 2729/3470 (respectively) and ligated to pTrcKX as described for the *E*. *coli* genes. The *ompC* genes from *S*. Typhimurium LT2 and *E*. *cloacae* were amplified with primers 3326/2388, digested with NcoI/XhoI and ligated to plasmid pTrc99a to generate pCH11852 and pCH11853, respectively. All *E*. *coli ompC* alleles were amplified with primer pair 2387/2388, followed by ligation to plasmid pTrcKX using KpnI and XhoI restriction sites. The coding sequences for OmpC^CFT073^ loops L4, L5, L7 and L8 of OmpC^CFT073^ were replaced using megaprimer PCR [61]. Plasmid pCH10778 template DNA was amplified with reverse primer 2388 in conjunction with primers 2935 (L5^EC536^), 2936 (L7^EC536^) and 2937 (L8^EC536^). The resulting products were used as megaprimers to amplify *ompC* ^CFT073^ in conjunction with forward primer 2387. The chimeric products were digested with KpnI/XhoI and ligated to pTrcKX to generate plasmids pCH11334, pCH11765 and pCH11335. The same procedure and primers were used to generate *ompC* ^CFT073^ constructs encoding two loops from *ompC* ^EC536^. The loop L4 exchange megaprimer was generated with primers 2387/2807 and used with 2388 to generate the final product. All PCR products were digested with KpnI and XhoI and ligated to plasmid pTrcKX.

**Table 2:** Plasmid constructs.

*Plasmids*	*Description* ^*a*^	*Reference*
pTrc99a	IPTG-inducible expression vector, Amp^R^	Amersham
pTrcKX	Derivative of pTrc99a with additional KpnI and XhoI restriction sites, Amp^R^	[74]
pCP20	temperature-inducible expression of FLP recombinase, Cm^R^ Amp^R^	[58]
pSC189*kan*	Mobilizable plasmid with R6Kγ replication origin. Carries the *mariner* transposon containing a kanamycin-resistance cassette, Amp^R^ Kan^R^	[63]
pWEB::TNC	pWEB::TNC (CDI^–^), Amp^R^, Cm^R^	Epicentre
pDAL660Δ1–39	Constitutively expresses the *E*. *coli* EC93 *cdiBAI* gene cluster, Amp^R^	[1]
pDAL866	Arabinose-inducible expression of the *E*. *coli* 536 *cdiBAI* gene cluster, Amp^R^ Cm^R^	[32]
pZS21	pSC101-derived plasmid vector, Kan^R^	[75]
pZS21*amp*	pZS21 derivative with ampicillin-resistance cassette, Amp^R^	[4]
pZS21*amp*-*bamA* ^Eco^	Constitutive expression of *E*. *coli bamA*, Amp^R^	[4]
pZS21*amp*-*bamA* ^ECL^	Constitutive expression of *E*. *cloacae bamA*, Amp^R^	[20]
pET21::*colE5-immE*5	Over-produces colicin E5 and ImmE5-His_6_, Amp^R^	[74]
pCH450	pACY184 derivative that carries *E*. *coli araC* and the L-arabinose-inducible P_BAD_ promoter. Tet^R^	[59]
pCH9181	pTrcKX::*cdiA-CT/cdiI* ^Dd3937^-*his*_*6*_, Amp^R^	[76]
pCH9763	pBR322::*cysK*, Amp^R^	This study
pCH10138	pTrcKX::*ompC* ^K-12^, Amp^R^	This study
pCH10201	pTrcKX::*ompC* ^F11^, Amp^R^	This study
pCH10202	pZS21*-ompF* ^K-12^, Kan^R^	This study
pCH11338	pTrcKX::*ompC* ^EC536^, Amp^R^	This study
pCH10458	pTrcKX::*ompC* ^EC869^, Amp^R^	This study
pCH10460	pTrcKX::*ompC* ^EC93^, Amp^R^	This study
pCH10778	pTrcKX::*ompC* ^CFT073^, Amp^R^	This study
pCH10779	pTrcKX::*ompC* ^UTI89^, Amp^R^	This study
pCH10780	pTrcKX::*ompF* ^EC536^, Amp^R^	This study
pCH10796	pTrcKX::*link8-ompF*, Amp^R^	This study
pCH10797	pTrc99a::*ompF*_2_, Amp^R^	This study
pCH10959	pTrcKX::*ompC*(L4^F11^)^CFT073^, Amp^R^	This study
pCH11330	pTrcKX::*ompC* ^6104H^, Amp^R^	This study
pCH11331	pTrcKX::*ompC* ^A54H^, Amp^R^	This study
pCH11332	pTrcKX::*ompC* ^A42H^, Amp^R^	This study
pCH11333	pTrcKX::*ompC* ^A35H^, Amp^R^	This study
pCH11341	pKAN-*micF-ompC* ^EC536^, Amp^R^ Kan^R^	This study
pCH11342	pKAN-*abpE´*, Amp^R^ Kan^R^	This study
pCH11496	pCH450::DsRed; arabinose-inducible expression of DsRed, Tet^R^	This study
pCH11627	pTrcKX::*ompC* ^A33H^, Amp^R^	This study
pCH11334	pTrcKX::*ompC*(L5^536^)^CFT073^, Amp^R^	This study
pCH11335	pTrcKX::*ompC*(L8^536^)^CFT073^, Amp^R^	This study
pCH11336	pTrcKX::*ompC*(L5/L7^536^)^CFT073^, Amp^R^	This study
pCH11337	pTrcKX::*ompC*(L7/L8^536^)^CFT073^, Amp^R^	This study
pCH11765	pTrcKX::*ompC*(L7^536^)^CFT073^, Amp^R^	This study
pCH11767	pTrcKX::ompC(L5/L8^536^)^CFT073^, Amp^R^	This study
pCH11850	pTrcKX::*ompF* ^K-12^, Amp^R^	This study
pCH11851	pTrcKX::*ompF* ^ECL^, Amp^R^	This study
pCH11852	pTrcKX::*ompF* ^LT2^, Amp^R^	This study
pCH11853	pTrcKX::*ompC* ^ECL^, Amp^R^	This study
pCH11854	pTrcKX::*ompC* ^LT2^, Amp^R^	This study
pCH12054	pTrc99a::*ompF*_2_*, Amp^R^	This study

^a^Abbreviations: Amp^R^, ampicillin resistant; Cm^R^, chloramphenicol resistant; Kan^R^, kanamycin resistant; Tet^R^, tetracycline resistant

Dimeric OmpF expression plasmids were constructed through sequential ligation of *ompF* coding sequences. The *ompF* ^EC536^ allele was amplified with primers 2733/2734 and ligated to pCH9181 using SpeI/XhoI restriction sites to generate intermediate construct pCH10796. A second *ompF* fragment was amplified with primers 2732/2729 and ligated to pCH10796 using KpnI/SpeI restriction sites. The resulting pCH10797 (*ompF*_2_) construct produces OmpF^EC536^ dimers linked by a Ser-Gly-Thr-Thr-Ser-Thr-Gly-Gly peptide. To introduce a longer linker sequence, *ompF* was amplified with primers 2729/2765 and ligated to pCH9181 using KpnI/SpeI sites to generate plasmid pCH11893. The second *ompF* module was amplified with primers 3008/2734 and ligated to pCH11893 using BamHI/XhoI restriction sites. The resulting pCH12054 (*ompF*_2_*) construct produces OmpF^EC536^ dimers linked by a Ser-Gly-Thr-Gly-Ser-Asp-Thr-Ser-Gly-Gly-Thr-Asp-Gly-Thr-Gly-Gly peptide.

### Competition co-cultures

Inhibitor and target bacteria were grown to mid-log phase in LB media supplemented with the appropriate antibiotics, then mixed at a 10:1 ratio in fresh LB medium without antibiotics and incubated for 4–5 h at 37°C with vigorous shaking in baffled flasks. Viable target-cell counts were enumerated on selective LB-agar as colony forming units (cfu) per mL. *E*. *coli* EPI100 carrying pDAL660Δ1–39 [1] or pDAL866 [33] were used as inhibitor cells for the comparison of CDI^EC93^ and CDI^EC536^ systems. The mock CDI^−^inhibitors were *E*. *coli* EPI100 carrying the pWEB::TNC vector. All other competitions used the *E*. *coli* 536 derivative DL6536, which expresses *cdiBAI* ^EC536^ under the control of an arabinose-inducible promoter [8]. The mock inhibitor strain DL6381 is a derivative of DL6536 that carries a deletion of the *cdiA-CT* coding sequence [8]. DL6536 and DL6381 were cultured in 0.2% arabinose to induce *cdiBAI* expression and competition co-cultures were supplemented with 0.2% arabinose.

### Transposon library construction and selection for CDI^R^ mutants

Plasmid pSC189 (encoding the *mariner* transposon) was introduced into *E*. *coli* CH10013 cells carrying pCH9763 (pBR322::*cysK*) through conjugation with *E*. *coli* MFD*pir* donors [62, 63]. Donor and recipient cells were grown to mid-log phase in LB media supplemented with ampicillin and 30 μM diaminopimelic acid (for donors). Donors (~6.0 ×10^8^ colony forming units, cfu) and recipients (~3 ×10^8^ cfu) were mixed and collected by centrifugation for 2 min at 9,000 rpm in a microcentrifuge. The supernatant was removed by aspiration and the cell pellet resuspended in 100 μL of 1× M9 salts. Cell mixtures were spotted onto 0.45 μm nitrocellulose membranes and incubated on LB-agar without inversion for 4 h at 37°C. Cells were then harvested into 2 mL of 1× M9 salts and transposon-insertion mutants selected on LB-agar supplemented with rifampicin and kanamycin. Over 50,000 colonies from each mating were harvested into 1× M9 salts, and inoculated into 50 mL of LB medium in a 250 mL baffled flask. Inhibitor cells (*E*. *coli* 536 derivative DL6536) were grown in parallel in LB medium supplemented with L-arabinose until mid-log phase. Inhibitors and targets were mixed at a 10:1 ratio in fresh LB medium supplemented with L-arabinose and cultured for 4 h with shaking at 37°C. Viable target cells were enumerated as cfu/mL on LB agar supplemented with rifampicin and kanamycin. Survivors from the first round of CDI^EC536^ selection were harvested into 1× M9 salts and inoculated into 50 mL of LB medium supplemented with L-arabinose for a second round of selection. After the third round of selection, target-cell populations were completely resistant to the CDI inhibitor cells. Individual colonies were screened for CysK activity in MOPS minimal media containing 3 mM 1,2,4-triazole, which inhibits the growth of *cysK* ^*+*^ bacteria [64]. CDI^R^ phenotypes for all *cysK* ^*+*^ clones were confirmed in competition co-cultures with *E*. *coli* 536 inhibitors. Transposon mutations were transduced into CDI-sensitive cells, and the resulting transductants tested in competition co-cultures for CDI^R^ phenotypes.

### Flow cytometry analysis of cell-cell adhesion

Overnight cultures of GFP-labeled *E*. *coli* DL4905 cells carrying pDAL660Δ1–39 (CDI^EC93^), pDAL866 (CDI^EC536^) or pWEB::TNC (CDI^−^) were diluted into fresh tryptone broth (TB) and grown to mid-log phase at 30°C. Inhibitor cells were then mixed at a 5:1 ratio with DsRed-labeled *E*. *coli* CH10099 (Δ*ompC*) or CH10100 (Δ*ompF*) cells complemented with the indicated *ompC* and *ompF* expressing plasmids. Cell suspensions were incubated with aeration for 15 min at 30°C, diluted 1:50 into filtered 1× phosphate-buffered saline (PBS), and then analyzed on an Accuri C6 flow cytometer using FL1 (533/30 nm, GFP) and FL2 (585/40 nm, DS-Red) fluorophore filters (Becton Dickinson) as described [36].

### Immunoblot analysis

Bacteria were grown to mid-log phase in LB media supplemented with ampicillin. Cells were collected by centrifugation at 6,000 ×*g*. Cell were frozen at –80°C, then broken by freeze-thaw cycles in 8 M urea, 150 mM NaCl, 50 mM Tris-HCl (pH 8.0). Extracts were quantified by Bradford assay and equal amounts of protein were resolved on 6 M urea/10% SDS polyacrylamide gels for 4 h at 100 V. Gels were electroblotted onto nitrocellulose membranes and OMPs detected using polyclonal antisera raised against *E*. *coli* OmpC/OmpF and BamA. Immunoblots were visualized using IRDye 680 (LI-COR) labeled anti-rabbit secondary antibodies and an Odyssey infrared imager.

### Colicin E5 purification and activity assays

Colicin E5 was purified in complex with its His_6_-tagged immunity protein as described previously [6, 65]. *E*. *coli* strains CH12180 and CH12345 carrying pCH11850 (*ompF*), pCH10796 (*ompF-ompF*, 8-residue linker), or pCH12054 (*ompF-ompF*, 16-residue linker) were grown to mid-log phase in LB media supplemented with ampicillin, then collected by centrifugation and re-suspended in fresh pre-warmed media at OD_600_ = 0.05. After 30 min of incubation with shaking, purified colicin E5 was added to a final concentration of 1 μM and cell growth monitored by measuring the OD_600_ every 60 min.

## Supporting Information

S1 Fig Alignment of OmpC sequences from selected *E*. *coli* isolates. The sequences of mature OmpC proteins were aligned using Clustal-Omega and identical residues indicated with asterisks (*). Extracellular loop sequences are shown in red and β-strands in boldface.
(PDF)

S2 Fig Inter-protomer interactions for OmpC^K-12^ and OmpF^K-12^. The sequences of mature OmpC^K-12^ and OmpF^K-12^ were aligned using Clustal-Omega and identical residues indicated with asterisks (*). Extracellular loops are shown in red font and β-strands are underlined. Buried interfacial residues are shown in blue, and residues involved in direct inter-protomer H-bonds and salt-bridges are in orange. Contacts were determined with PDBePISA using PDB:2J1N (OmpC^K-12^) and PDB:3POX (OmpF^K-12^).
(PDF)

S3 Fig Inter-protomer contacts are conserved between OmpC^K-12^ and OmpF^K-12^. Individual protomers of (A) OmpC^K-12^ (PDB:2J1N) and (B) OmpF^K-12^ (PDB:3POX) are viewed from the inter-protomer interface (left) and from the extracellular milieu (right). Extracellular loops L2 and L4 from adjacent protomers are shown as yellow sticks and β-strands involved inter-subunit contacts are labeled in the left panels. Buried interfacial residues are shown in blue, and residues involved in direct inter-protomer H-bonds and salt-bridges are in orange. Contacts were determined with PDBePISA. The conserved inter-subunit ion-pairs are shown in the right panels with residues labeled.
(TIF)

S4 Fig Alignment of CdiA^EC93^ and CdiA^EC536^. CdiA^EC93^ and CdiA^EC536^ sequences were aligned using Clustal-Omega and identical residues are highlighted in blue. Predicted domains and peptide motifs are as described by the InterPro web server. CdiA^EC93^ annotations are available at http://www.ebi.ac.uk/interpro/protein/Q3YL96, and CdiA^EC536^ at http://www.ebi.ac.uk/interpro/protein/Q0T963. The signal sequence and TPS transport domain are required for CdiA export. FHA-1 (Pfam: PF05594) and FHA-2 (PF13332) peptide repeats are predicted to form a β-helix. The pretoxin-VENN domain (PT-VENN; PF04829) demarcates the variable C-terminal toxin region.
(TIF)

S5 Fig Comparison of *E*. *coli* OmpC structures. Individual OmpC protomers from *E*. *coli* K-12 (PDB:2J1N), CFT073 (PDB:2XE1) and 6104H (PDB:2XE2) are shown in surface representation as viewed from the extracellular milieu. Extracellular loops L4 (maroon), L5 (green) and L7 (orange) are labeled in the top row of images. The bottom row shows the OmpC protomers in the same orientation, but with the surface rendered as an electrostatic potential map. Red areas are electronegative, blue are electropositive and white are neutral.
(TIF)

S1 Table Predicted OmpC proteins from *E*. *coli* isolates. OmpC from *E*. *coli* str. K-12 substr. MG1655 was used to query all *E*. *coli* isolates using BLASTP 2.2.32+ at https://blast.ncbi.nlm.nih.gov/Blast.cgi. Proteins with identical amino acid sequences are grouped with colored backgrounds. Extracellular loop L4, L5 and L7 variations are indicated according the scheme outlined in S2 Table. Asterisks (*) indicate OmpC variants tested in this study.
(XLSX)

S2 Table *E*. *coli* OmpC loop L4, L5 and L5 sequences. Loop sequences were determined for 2707 predicted *E*. *coli* OmpC proteins. Sequences were grouped and numbered according to sequence identity. Red residues are shared with OmpC from *E*. *coli* K-12.
(XLSX)

S3 Table OmpC and OmpF interprotomer H-bonds and salt-bridges. Predicted hydrogen-bonds (H-bonds) and salt-bridges between individual protomers of *E*. *coli* K-12 OmpC (PDB: 2J1N) and OmpF (PDB: 3POX) were determined using PDBePISA (http://www.ebi.ac.uk/pdbe/pisa/). Solvent accessible surface areas (ASA) and buried surface areas (BSA) were determined using PDBePISA (http://www.ebi.ac.uk/pdbe/pisa/). Residues highlighted in orange are buried at interprotomer interfaces. Predicted hydrogen bonds are indicated by H and salt-bridges by SH.
(XLSX)

S4 Table OmpC intraprotomer loop-loop interactions. Extracellular loop residues involved in intraprotomer loop-loop interactions are presented for three *E*. *coli* OmpC proteins. Loop L4, L5 and L7 sequences are labeled according to the scheme outlined in S2 Table.
(XLSX)

S5 Table Oligonucleotides used in this study. (DOCX)
