# Supplementary material for: CdiA Effectors from Uropathogenic Escherichia coli Use Heterotrimeric Osmoporins as Receptors to Recognize Target Bacteria
Source: PLoS Pathog. 2016 Oct 10;12(10):e1005925. doi: 10.1371/journal.ppat.1005925 (PMC5056734; doi:10.1371/journal.ppat.1005925)
Supplement: S1 Fig — The sequences of mature OmpC proteins were aligned using Clustal-Omega and identical residues indicated with asterisks (*). Extracellular loop sequences are shown in red and β-strands in boldface. (PDF) [file ppat.1005925.s001.pdf]

```
          β1          β2          L1        β3                β4
K12     AEVYNKDGNKLDLYGKVDGLHYFSDNKDVDGDQTYMRLGFKGETQVTDQLTGYGQWEYQI 60
EC536   AEVYNKDGNKLDLYGKVDGLHYFSDDKSVDGDQTYMRLGFKGETQVTDQLTGYGQWEYQI 60
CFT073  AEVYNKDGNKLDLYGKVDGLHYFSDDKSVDGDQTYMRLGFKGETQVTDQLTGYGQWEYQI 60
A35H    AEVYNKDGNKLDLYGKVDGLHYFSDDKSVDGDQTYMRLGFKGETQVTDQLTGYGQWEYQI 60
6104H   AEIYNKDGNKLDLYGKVDGLHYFSDNDSKDGDKTYMRLGFKGETQVTDQLTGYGQWEYQI 60
A54H    AEVYNKDGNKLDLYGKVDGLHYFSDNKSEDGDQTYVRLGFKGETQVTDQLTGYGQWEYQI 60
A42H    AEVYNKDGNKLDLYGKVDGLHYFSDDKSVDGDQTYMRLGFKGETQVTDQLTGYGQWEYQI 60
F11     AEVYNKDGNKLDLYGKVDGLHYFSDDKSVDGDQTYMRLGFKGETQVTDQLTGYGQWEYQI 60
EC93    AEVYNKDGNKLDLYGKVDGLHYFSDNKDVDGDQTYMRLGFKGETQVTDQLTGYGQWEYQI 60
EC869   AEVYNKDGNKLDLYGKVDGLHYFSDDKSVDGDQTYMRLGFKGETQVTDQLTGYGQWEYQI 60
UTI89   AEVYNKDGNKLDLYGKVDGLHYFSDDKSVDGDQTYMRLGFKGETQVTDQLTGYGQWEYQI 60
A33H    AEVYNKDGNKLDLYGKVDGLHYFSDDKSVDGDQTYMRLGFKGETQVTDQLTGYGQWEYQI 60
        **:**********************:.. ***:**:************************

               L2        β5          β6                   L3
K12     QGNSAE-NENNSWTRVAFAGLKFQDVGSFDYGRNYGVVYDVTSWTDVLPEFGGDTYGSDN 119
EC536   QGNAPE-SENNSWTRVAFAGLKFQDIGSFDYGRNYGVVYDVTSWTDVLPEFGGDTYGSDN 119
CFT073  QGNAPE-SENNSWTRVAFAGLKFQDIGSFDYGRNYGVVYDVTSWTDVLPEFGGDTYGSDN 119
A35H    QGNAPE-SENNSWTRVAFAGLKFQDVGSFDYGRNYGVVYDVTSWTDVLPEFGGDTYGSDN 119
6104H   QGNEPE-SDNSSWTRVAFAGLKFQDVGSFDYGRNYGVVYDVTSWTDVLPEFGGDTYDSDN 119
A54H    QGNTSEDNKENSWTRVAFAGLKFQDVGSFDYGRNYGVVYDVTSWTDVLPEFGGDTYGSDN 120
A42H    QGNSAE-NENNSWTRVAFAGLKFQDVGSFDYGRNYGVVYDVTSWTDVLPEFGGDTYGSDN 119
F11     QGNSAE-NENNSWTRVAFAGLKFQDVGSFDYGRNYGVVYDVTSWTDVLPEFGGDTYGSDN 119
EC93    QGNSAE-NENNSWTRVAFAGLKFQDVGSFDYGRNYGVVYDVTSWTDVLPEFGGDTYGSDN 119
EC869   QGNSAE-NENNSWTRVAFAGLKFQDVGSFDYGRNYGVVYDVTSWTDVLPEFGGDTYGSDN 119
UTI89   QGNSAE-NENNSWTRVAFAGLKFQDVGSFDYGRNYGVVYDVTSWTDVLPEFGGDTYGSDN 119
A33H    QGNSAE-NENNSWTRVAFAGLKFQDVGSFDYGRNYGVVYDVTSWTDVLPEFGGDTYGSDN 119
        ***  *  ..:.**************:****************************** ***

                    β7              β8                  L4
K12     FMQQRGNGFATYRNTDFFGLVDGLNFAVQYQGKNGNPSGEGFT----SGVTNNGR-DALR 174
EC536   FMQQRGNGFATYRNTDFFGLVDGLNFAVQYQGQNGSVSGENDPDFTGHGITNNGR-KALR 178
CFT073  FMQQRGNGFATYRNTDFFGLVDGLNFAVQYQGQNGSVSGENDPDFTGHGITNNGR-KALR 178
A35H    FMQQRGNGFATYRNTDFFGLVDGLNFAVQYQGQNGSVSGENDPDFTGHGITNNGR-KALR 178
6104H   FMQQRGNGFATYRNTDFFGLVDGLDFAVQYQGKNGSAHGEG--------MTTNGRDDVFE 171
A54H    FMQQRGNGFATYRNTDFFGLVDGLNFAVQYQGKNGSVSGEG--------MTNNGR-GALR 171
A42H    FMQQRGNGFATYRNTDFFGLVDGLNFAVQYQGKNGSVDGEG--------MTNNGR-GALR 170
F11     FMQQRGNGFATYRNTDFFGLVDGLNFAVQYQGKNGSVSGEG--------MTNNGR-DALR 170
EC93    FMQQRGNGFATYRNTDFFGLVDGLNFAVQYQGKNGSVSGEG--------MTNNGR-GALR 170
EC869   FMQQRGNGFATYRNTDFFGLVDGLNFAVQYQGKNGSVSGEG--------MTNNGR-EALR 170
UTI89   FMQQRGNGFATYRNTDFFGLVDGLNFAVQYQGKNGSVSGEG--------MTNNGR-GALR 170
A33H    FMQQRGNGFATYRNTDFFGLVDGLNFAVQYQGKNGSVSGEG--------MTNNGR-GALR 170
        ************************:*******:**.  **         :*.***  .:.

               β9             β10             L5               β11
K12     QNGDGVGGSITYDYEGFGIGGAISSSKRTDAQN--------TAAYIGNGDRAETYTGGLK 226
EC536   QNGDGVGGSITYDYEGFGVGAAVSSSKRTDAQN--------TAAYIGNGDRAETYTGGLK 230
CFT073  QNGDGVGGSITYDYEGFGVGAAVSSSKRTWDQNN--------TGLIGTGDRAETYTGGLK 230
A35H    QNGDGVGGSITYDYEGFGIGGAISSSKRTWDQNN--------TGLIGTGDRAETYTGGLK 230
6104H   QNGDGVGGSITYNYEGFGIGAAVSSSKRTWDQNN--------TGLIGTGDRAETYTGGLK 223
A54H    QNGDGVGGSITYDYEGFGIGAAVSSSKRTDDQNGSYISNGVVRNYIGTGDRAETYTGGLK 231
A42H    QNGDGVGGSITYDYEGFGVGAAVSSSKRTDAQNGTYVVDNVTHNYIGTGDRAETYTGGLK 230
F11     QNGDGVGGSITYDYEGFGIGGAISSSKRTDAQN--------TAAYIGNGDRAETYTGGLK 222
EC93    QNGDGVGGSITYDYEGFGIGGAISSSKRTDDQN--------SPLYIGNGDRAETYTGGLK 222
EC869   QNGDGVGGSITYDYEGFGIGAAVSSSKRTDDQN--------SPLYIGNGDRAETYTGGLK 222
UTI89   QNGDGVGGSITYDYEGFGIGGAISSSKRTDDQN--------SPLYIGNGDRAETYTGGLK 222
A33H    QNGDGVGGSITYDYEGFGIGGAISSSKRTDDQN--------SPLYIGNGDRAETYTGGLK 222
        ************:*****:*.*:******  **            **.************
```

```
                    β12          L6        β13              β14
K12       YDANNIYLAAQYTQTYNATRVGSLGWANKAQNFEAVAQYQFDFGLRPSLAYLQSKGKNLG 286
EC536     YDANNIYLAAQYTQTYNATRVGSLGWANKAQNFEAVAQYQFDFGLRPSVAYLQSKGKNLG 290
CFT073    YDANNIYLAAQYTQTYNATRVGSLGWANKAQNFEAVAQYQFDFGLRPSVAYLQSKGKNLG 290
A35H      YDANNIYLAAQYTQTYNATRVGSLGWANKAQNFEAVAQYQFDFGLRPSVAYLQSKGKNLG 290
6104H     YDANNIYLAAQYTQTYNATRVGSLGWANKAQNFEAVAQYQFDFGLRPSLAYLQSKGKNLG 283
A54H      YDANNIYLAAQYTQTYNATRVGSLGWANKAQNFEAVAQYQFDFGLRPSVAYLQSKGKNLG 291
A42H      YDANNIYLAAQYTQTYDATRVGSLGWANKAQNFEAVAQYQFDFGLRPSLAYLQSKGKNLG 290
F11       YDANNIYLAAQYTQTYNATRVGSLGWANKAQNFEAVAQYQFDFGLRPSVAYLQSKGKNLG 282
EC93      YDANNIYLAAQYTQTYNATRVGSLGWANKAQNFEAVAQYQFDFGLRPSVAYLQSKGKNLG 282
EC869     YDANNIYLAAQYTQTYNATRVGSLGWANKAQNFEAVAQYQFDFGLRPSLAYLQSKGKNLG 282
UTI89     YDANNIYLAAQYTQTYNATRVGSLGWANKAQNFEAVAQYQFDFGLRPSLAYLQSKGKNLG 282
A33H      YDANNIYLAAQYTQTYNATRVGSLGWANKAQNFEAVAQYQFDFGLRPSVAYLQSKGKNLG 282
          ****************:*******************************:***********

               L7           β15            β16          L8          β1´
K12       ----RGYDDEDILKYVDVGATYYFNKNMSTYVDYKINLLDDNQFTRDAGINTDNIVALGL 342
EC536     TIGTRNYDDEDILKYVDVGATYYFNKNMSTYVDYKINLLDDNQFTRDAGINTDNIVALGL 350
CFT073    VVAGRNYDDEDILKYVDVGATYYFNKNMSTYVDYKINLLDDNQFTRAAGINTDDIVALGL 350
A35H      VINGRNYDDEDILKYVDVGATYYFNKNMSTYVDYKINLLDDNQFTRAAGINTDDIVALGL 350
6104H     ----RGYDDEDILKYVDVGATYYFNKNMSTYVDYKINLLDDNRFTRDAGINTDDIVALGL 339
A54H      VINGRNYDDEDILKYVDVGATYYFNKNMSTYVDYKINLLDDNQFTRDAGINTDNIVALGL 351
A42H      VINGRNYDDEDILKYVDVGATYYFNKNMSTDVDYKINLLDDNQFTRDAGINTDNIVALGL 350
F11       TIGTRNYDDEDILKYVDVGATYYFNKNMSTYVDYKINLLDDNQFTRDAGINTDNIVALGL 342
EC93      VINGRNYDDEDILKYVDVGATYYFNKNMSTYVDYKINLLDDNQFTRDAGINTDNIVALGL 342
EC869     VINGRNYDDEDILKYVDVGATYYFNKNMSTYVDYKINLLDDNQFTRDAGINTDNIVALGL 342
UTI89     ----RGYDDEDILKYVDVGATYYFNKNMSTYVDYKINLLDDNQFTRDAGINTDNIVALGL 338
A33H      TIAGRNYDDEDILKYVDVGATYYFNKNMSTYVDYKINLLDDNQFTRDAGINTDNIVALGL 342
              * ************************ ***********:*** ******:******

K12       VYQF 346
EC536     VYQF 354
CFT073    VYQF 354
A35H      VYQF 354
6104H     VYQF 343
A54H      VYQF 355
A42H      VYQF 354
F11       VYQF 346
EC93      VYQF 346
EC869     VYQF 346
UTI89     VYQF 342
A33H      GYQF 346
           ***
```
