# Supplementary material for: CdiA Effectors from Uropathogenic Escherichia coli Use Heterotrimeric Osmoporins as Receptors to Recognize Target Bacteria
Source: PLoS Pathog. 2016 Oct 10;12(10):e1005925. doi: 10.1371/journal.ppat.1005925 (PMC5056734; doi:10.1371/journal.ppat.1005925)
Supplement: S2 Fig — The sequences of mature OmpCK-12 and OmpFK-12 were aligned using Clustal-Omega and identical residues indicated with asterisks (*). Extracellular loops are shown in red font and β-strands are underlined. Buried interfacial residues are shown in blue, and residues involved in direct inter-protomer H-bonds and salt-bridges are in orange. Contacts were determined with PDBePISA using PDB:2J1N (OmpCK-12) and PDB:3POX (OmpFK-12). (PDF) [file ppat.1005925.s002.pdf]

```
       β1          β2            L1           β3              β4
OmpC AEVYNKDGNKLDLYGKVDGLHYFSDNKD-----VDGDQTYMRLGFKGETQVTDQLTGYGQ  55
OmpF AEIYNKDGNKVDLYGKAVGLHYFSKGNGENSYGGNGDMTYARLGFKGETQINSDLTGYGQ  60
     **:*******:*****. ******. :       :** ** *********:..:******

                L2          β5           β6             L3
OmpC WEYQIQGNSAENE---NNSWTRVAFAGLKFQDVGSFDYGRNYGVVYDVTSWTDVLPEFGG  112
OmpF WEYNFQGNNSEGADAQTGNKTRLAFAGLKYADVGSFDYGRNYGVVYDALGYTDMLPEFGG  120
     ***::***.:*     . . **:******: ****************. .:**:******

                       β7              β8              L4
OmpC DTYGSDNFMQQRGNGFATYRNTDFFGLVDGLNFAVQYQGKNGNPSGEGFTSGVTNNGRDA  172
OmpF DTAYSDDFFVGRVGGVATYRNSNFFGLVDGLNFAVQYLGKNERDT--------------A  166
     **  **:*:  *  *.*****::************** *** .                *

              β9           β10          L5            β11
OmpC LRQNGDGVGGSITYDYEGFGIGGAISSSKRTDAQNTAAYIGNGDRAETYTGGLKYDANNI  232
OmpF RRSNGDGVGGSISYEYEGFGIVGAYGAADRTNLQE-AQPLGNGKKAEQWATGLKYDANNI  225
      *.*********:*:****** ** .::.**: *: *  :***.:** :: *********

        β12            L6            β13            β14
OmpC YLAAQYTQTYNATRV-----GSLGWANKAQNFEAVAQYQFDFGLRPSLAYLQSKGKNLGR  287
OmpF YLAANYGETRNATPITNKFTNTSGFANKTQDVLLVAQYQFDFGLRPSIAYTKSKAKDV-E  284
     ****:* :* *** :      : *:***:*:.  *************:** :**.*:: .

      L7      β15              β16         L8            β1´
OmpC GYDDEDILKYVDVGATYYFNKNMSTYVDYKINLLDDNQFTRDAGINTDNIVALGLVYQF  346
OmpF GIGDVDLVNYFEVGATYYFNKNMSTYVDYIINQIDSD---NKLGVGSDDTVAVGIVYQF  340
     *  * *:::*.:***************** ** :*.:   .. *: :*: **:*:****
```
