# Supplementary figures and images for: CdiA Effectors from Uropathogenic Escherichia coli Use Heterotrimeric Osmoporins as Receptors to Recognize Target Bacteria
Source: PLoS Pathog. 2016 Oct 10;12(10):e1005925. doi: 10.1371/journal.ppat.1005925 (PMC5056734; doi:10.1371/journal.ppat.1005925)

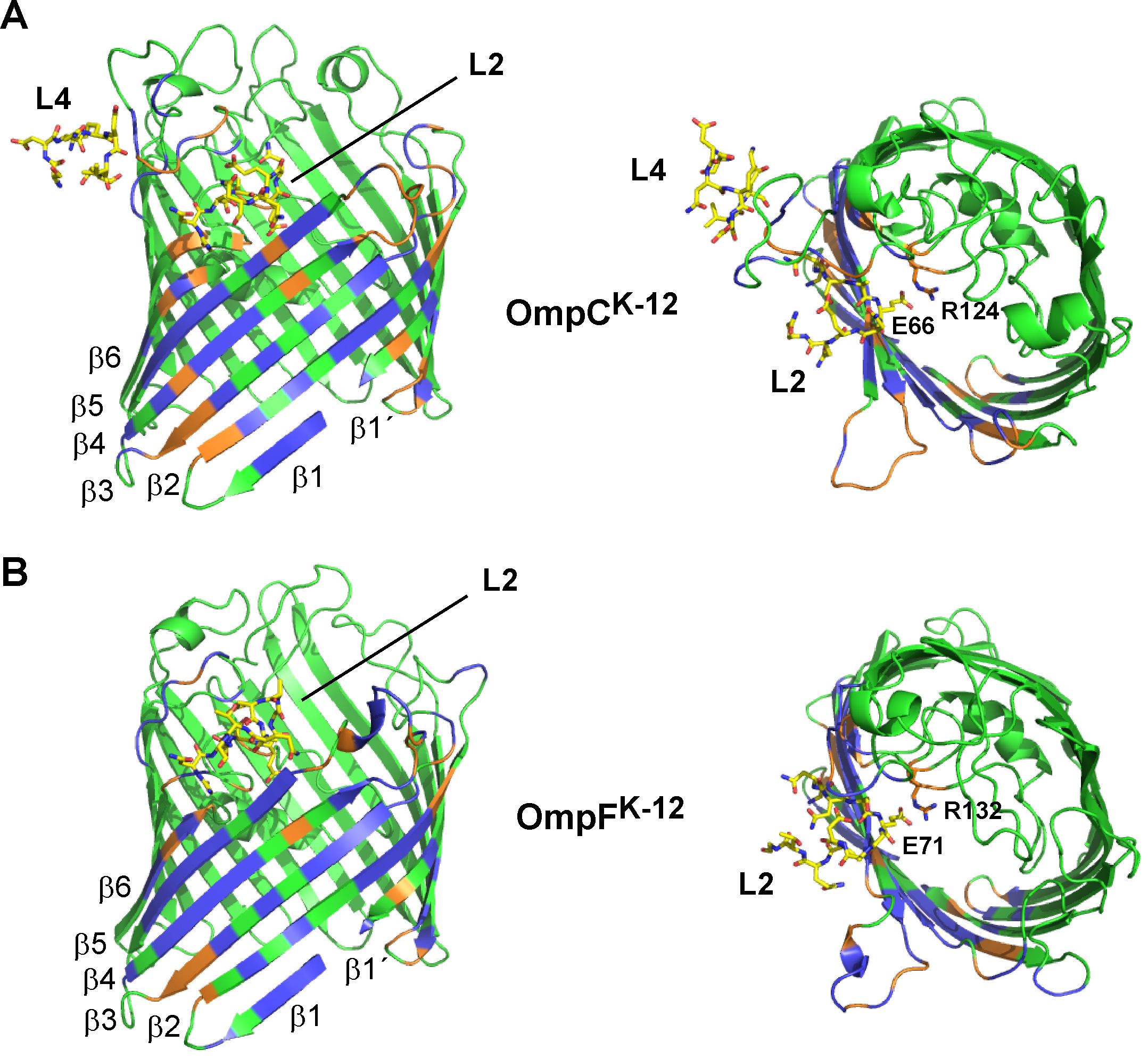

Supplement: S3 Fig — Individual protomers of (A) OmpCK-12 (PDB:2J1N) and (B) OmpFK-12 (PDB:3POX) are viewed from the inter-protomer interface (left) and from the extracellular milieu (right). Extracellular loops L2 and L4 from adjacent protomers are shown as yellow sticks and β-strands involved inter-subunit contacts are labeled in the left panels. Buried interfacial residues are shown in blue, and residues involved in direct inter-protomer H-bonds and salt-bridges are in orange. Contacts were determined with PDBePISA. The conserved inter-subunit ion-pairs are shown in the right panels with residues labeled. (TIF) [file ppat.1005925.s003.TIF]

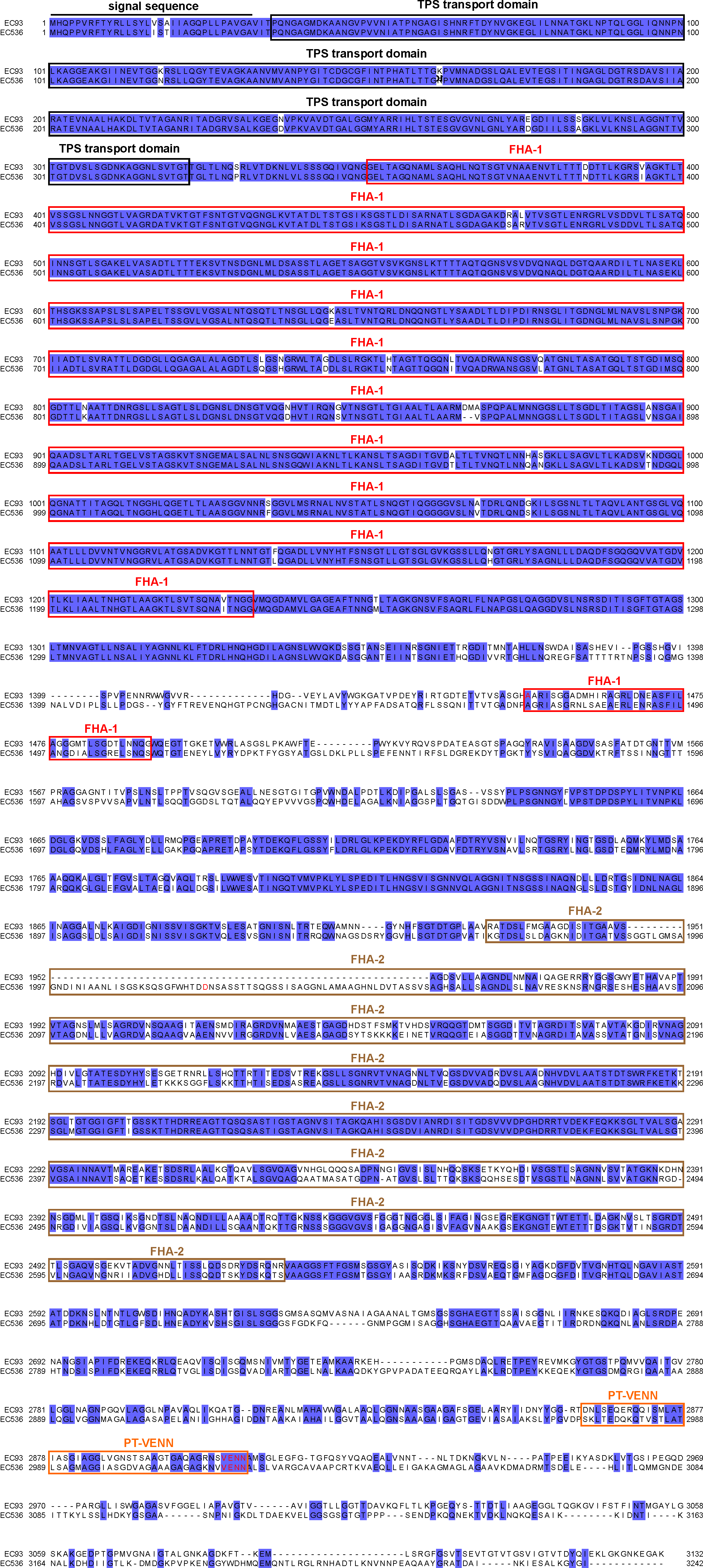

Supplement: S4 Fig — CdiAEC93 and CdiAEC536 sequences were aligned using Clustal-Omega and identical residues are highlighted in blue. Predicted domains and peptide motifs are as described by the InterPro web server. CdiAEC93 annotations are available at http://www.ebi.ac.uk/interpro/protein/Q3YL96, and CdiAEC536 at http://www.ebi.ac.uk/interpro/protein/Q0T963. The signal sequence and TPS transport domain are required for CdiA export. FHA-1 (Pfam: PF05594) and FHA-2 (PF13332) peptide repeats are predicted to form a β-helix. The pretoxin-VENN domain (PT-VENN; PF04829) demarcates the variable C-terminal toxin region. (TIF) [file ppat.1005925.s004.TIF]

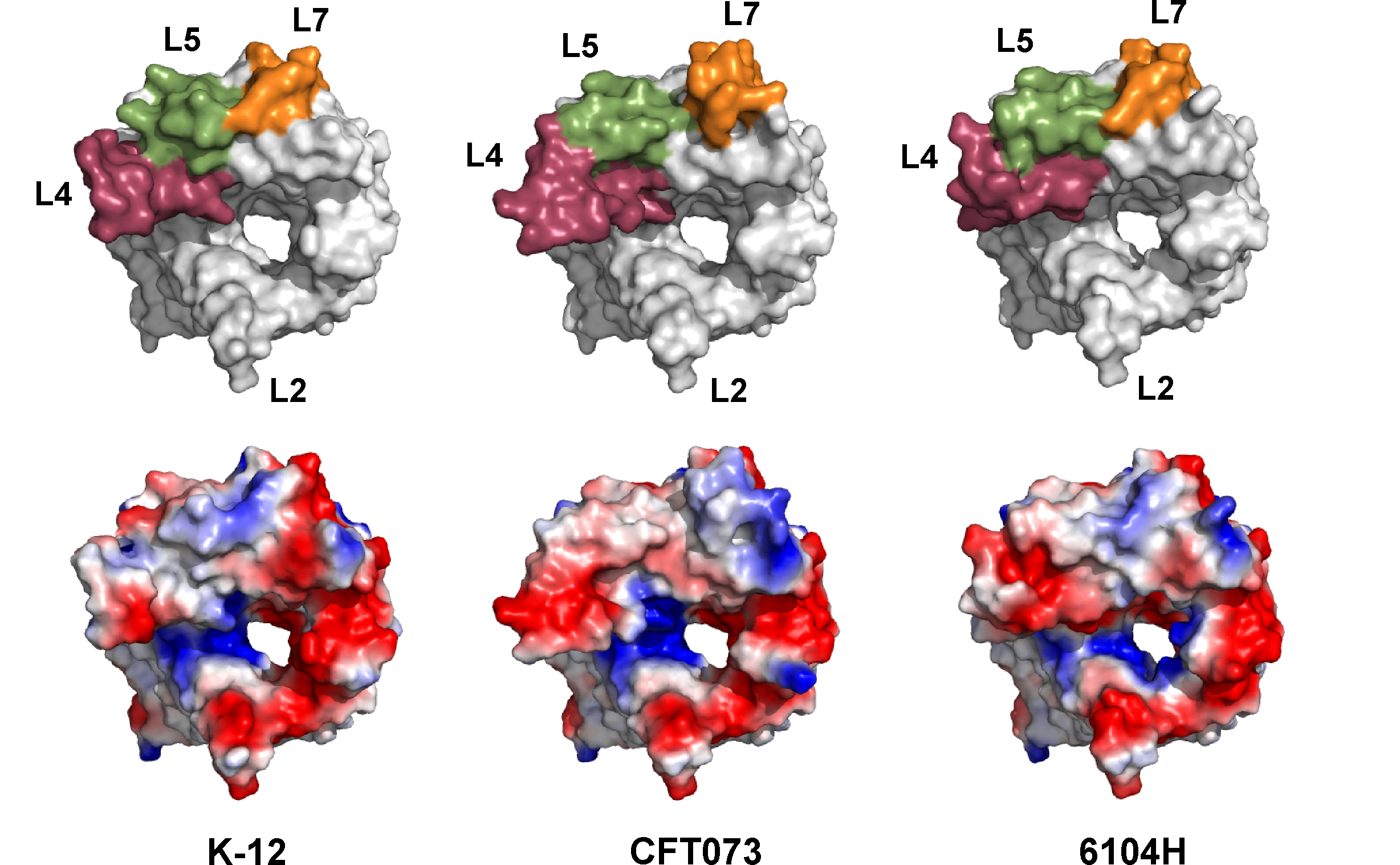

Supplement: S5 Fig — Individual OmpC protomers from E. coli K-12 (PDB:2J1N), CFT073 (PDB:2XE1) and 6104H (PDB:2XE2) are shown in surface representation as viewed from the extracellular milieu. Extracellular loops L4 (maroon), L5 (green) and L7 (orange) are labeled in the top row of images. The bottom row shows the OmpC protomers in the same orientation, but with the surface rendered as an electrostatic potential map. Red areas are electronegative, blue are electropositive and white are neutral. (TIF) [file ppat.1005925.s005.TIF]
