# Supplementary material for: CdiA Effectors from Uropathogenic Escherichia coli Use Heterotrimeric Osmoporins as Receptors to Recognize Target Bacteria
Source: PLoS Pathog. 2016 Oct 10;12(10):e1005925. doi: 10.1371/journal.ppat.1005925 (PMC5056734; doi:10.1371/journal.ppat.1005925)
Supplement: S5 Table — (DOCX) [file ppat.1005925.s010.docx]

**S5 Table. Oligonucleotides used in this study.**

| **Number** | **Oligonucleotide** | **Sequence** |
| --- | --- | --- |
| 2110 | cysK-Bam-rev | 5´ - GCT GGA TCC ATG CCA GGA GGG GCG AAA G |
| 2111 | cysK-EcoRV-for | 5´ - TCC CGA TAT CAT ACA GTT AAG GAC AGG C |
| 2385 | ompF-Bam-for | 5´ - TTT GGA TCC ATG AGG GTA ATA AAT AAT GAT G |
| 2386 | ompF-Xba-rev | 5´ - TTT TCT AGA GGT GTG CTA TTA GAA CTG |
| 2387 | ompC-Kpn-for | 5´ - GTT GGT ACC ATG AAA GTT AAA GTA CTG TCC |
| 2388 | ompC-Xho-rev | 5´ - TCA CTC GAG ATT AGA ACT GGT AAA CCA GAC |
| 2729 | ompF-K12-Kpn-for | 5´ - TTT GGT ACC ATG ATG AAG CGC AAT ATT C |
| 2732 | ompF-Spe-link-rev | 5´ - TTT ACT AGT AGT GCC GGA GAA CTG GTA AAC GAT AC |
| 2733 | ompF-Spe-link-for | 5´ - TTT ACT AGT ACC GC GGC GCA GAA ATC TAT AAC AAA GAT G |
| 2734 | ompF-K12-Xho-rev | 5´ - TTT CTC GAG TTA GAA CTG GTA AAC GAT ACC C |
| 2765 | ompF-Bam/Spe-rev | 5´ - TTT ACT AGT AGT GGA TCC AGT GCC GGA GAA CTG GTA AAC GAT AC |
| 2807 | CFT073-ompC(F11L4)-rev | 5´ - CTT TAC GAC CAT TGT TGG TCA TGC CTT CAC CGC TTA CGC TGC C |
| 2935 | CFT073-ompC(536L5)-for | 5´ - GCT CCA AAC GTA CCG ATG CTC AGA ACA CCG CTG CTT ACA TAG GCA ACG GCG ACC GTG CTG |
| 2936 | CFT073-ompC(536L7)-for | 5´ - AGG TAA AAA CCT GGG TAC TAT CGG TAC TCG TAA CTA CGA CGA C |
| 2937 | CFT073-ompC(536L8)-for | 5´ - CCA GTT CAC CCG CGA CGC TGG CAT CAA CAC TGA TAA CAT CGT AGC TC |
| 3008 | ompF-Bam-link-for | 5´ - TTT GGA TCC GAT ACC AGC GGA GGA ACA GAC GGA ACC GGC GGC G |
| 3320 | ompC(UPECswap)-Sac | 5´ - TTT GAG CTC CTG GAA ATT ATG CGG ATG |
| 3321 | ompC(UPECswap)-OE-rev | 5´ - GAC AGT ACT TTA ACT TTC ATG TTA TTA ACC CTC TGT TAT ATG |
| 3322 | ompC(UPECswap)-OE-for | 5´ - CAT ATA ACA GAG GGT TAA TAA CAT GAA AGT TAA AGT ACT GTC |
| 3323 | ompC(UPECswap)-Bam | 5´ - TTT GGA TCC TTA GAA CTG GTA AAC CAG |
| 3324 | ompC(UPECswap)-Eco | 5´ - TTT GAA TTC TCT CGA TTG ATA TCG AAC |
| 3325 | ompC(UPECswap)-Kpn | 5´ - TTT GGT ACC CCA CAG ATT CAC CAG C |
| 3326 | ompC-Nco-for | 5´ - TTT CCA TGG TAG TTA AAG TAC TGT CCC |
| 3468 | LT2-ompF-Kpn-for | 5´ - TTT GGT ACC ATG AAA CTT AAG TTA GTG GCA G |
| 3469 | LT2-ompF-Xho-rev | 5´ - TTT CTC GAG ATT AGA ACT GGT AGT TCA GAC C |
| 3470 | ECL-ompF-Xho-rev | 5´ - TTT CTC GAG ATT AGA ACT GGT AAA CCA GAC |
| 3504 | UPEC-ompC-OL-for | 5´ - CCA GTT CTA AGG ATC CCC CTG TGT AG |
| 3505 | UPEC-ompC-OL-rev | 5´ - GGG ATC CTT AGA ACT GGT AAA CCA G |
